# Effects of gender-affirming hormone therapy on body fat: a retrospective case‒control study in Chinese transwomen

**DOI:** 10.1186/s12944-024-02131-y

**Published:** 2024-05-17

**Authors:** Qin Pei, Yuwei Song, Zhongwei Huang, Hongkun Yu, Hao Xu, Xunda Ye, Lvfen Gao, Jian Gong, Xiaoying Tian

**Affiliations:** 1 School of Nursing, Jinan University, No. 601, West Huangpu Avenue, Guangzhou, Guangdong 510632 China; 2 Department of Obstetrics and Gynecology, The First Affiliated Hospital of Jinan University, No. 613, West Huangpu Avenue, Guangzhou, Guangdong 510630 China; 3 Department of Obstetrics and Gynaecology, National University Health Systems, Singapore, Singapore; 4 Department of Nuclear Medicine, The First Affiliated Hospital of Jinan University, No. 613, West Huangpu Avenue, Guangzhou, Guangdong 510630 China; 5 Central Laboratory, The First Affiliated Hospital of Jinan University, Guangzhou, China

**Keywords:** Fat, Transwomen, Gender-affirming hormone therapy, Lipids

## Abstract

**Background:**

There is insufficient research on how gender-affirming hormone therapy (GAHT) affects body fat modifications in transwomen from China. It is unclear whether hormone therapy affects the prevalence of obesity and blood lipid levels within this population. The current research aimed to assess how GAHT and treatment duration had an impact on the change in and redistribution of body fat in Chinese transwomen.

**Methods:**

This study included 40 transwomen who had not received GAHT and 59 who had. Body fat, blood lipid, and blood glucose levels were measured. GAHT is mainly a pharmacologic (estrogen and anti-androgen) treatment. The study also stratified participants based on the duration of GAHT to assess its impact on body fat distribution. The duration of GAHT was within one year, one to two years, two to three years, or more than three years.

**Results:**

After receiving GAHT, total body fat increased by 19.65%, and the percentage of body fat increased by 17.63%. The arm, corrected leg, and leg regions showed significant increases in fat content (+ 24.02%, + 50.69%, and + 41.47%, respectively) and percentage (+ 25.19%, + 34.90%, and + 30.39%, respectively). The total visceral fat content decreased (-37.49%). Based on the diagnostic standards for a body mass index ≥ 28 or total body fat percentage ≥ 25% or 30%, the chance of developing obesity did not change significantly. Blood glucose levels significantly increased (+ 12.31%). Total cholesterol levels (-10.45%) decreased significantly. Fat changes in those who received GAHT for one to two years were significantly different from those who did not receive GAHT.

**Conclusion:**

After receiving GAHT, total body fat and regional fat increased in Chinese transwomen, and the body fat distribution changed from masculine to feminine, especially during the first two years. However, neither the increase in total body fat percentage nor the decrease in visceral fat content didn’t bring about significant changes in the incidence of obesity, nor did triglycerides or low-density lipoprotein-cholesterol.

**Supplementary Information:**

The online version contains supplementary material available at 10.1186/s12944-024-02131-y.

## Background

Transwomen are individuals who are assigned biologically male at birth, but they strongly desire to transition their gender identity to female [1, 2]. To match their physical and social gender with their preferred female identity, transwomen often opt for gender-affirming hormone therapy (GAHT) and gender confirmation surgery (GCS). With or without GCS, GAHT are commonly pursued by transwomen to modify their physical appearance and body shape to match their desired gender identity [3, 4]. GAHT promotes the transition from a male physique to the ideal female body shape by causing a rise in total fat content in the body, a loss of fat-free mass, and a redistribution in body fatty tissues [5].

There are limited data available regarding alterations in body composition following GAHT in transwomen. Tack et al. [6] reported that, on average, transwomen lost 2.2 kg of lean body mass and gained 1.5 kg of body fat after an average of 10.6 months of GAHT. Numerous European studies have reported similar results [7–10]. According to prospective research by Yun et al. [11], there was a notable increase in regional body fat after 6 months of GAHT (27.4% in the leg area, 18% in the trunk area, and 27.2% in the gynoid area), leading to a more “feminized” distribution of body fat. Therefore, it is hypothesized that transwomen who receive GAHT will have increased fat in their bodies and decreased fat-free mass. Nevertheless, there is limited understanding of the magnitude of alterations in body fat and the point at which fat redistribution reaches a state of stability following long-term GAHT. Furthermore, there has not been any research on how GAHT affects Chinese transwomen’s body composition. Given these results, more research is required to improve our knowledge of how GAHT affects body composition, especially in Chinese transwomen. In addition, alterations in body composition might affect energy metabolism. For example, adipocytes are barely able to respond correctly to insulin signals in a state of body obesity, which leads to elevated blood glucose and blood lipids [12]. However, it is not clear from existing studies whether changes in blood lipid and glucose levels caused by hormone-induced body composition changes can increase the likelihood of developing cardiovascular disease (CVD) [13, 14].

This study retrospectively analyzed the body composition data of transwomen who did and did not receive GAHT at a tertiary hospital in Guangzhou, China. The aims of this study were to find out the effects of GAHT on transwomen’s total and regional body fat, as well as the evolution patterns of body fat during various treatment durations. Additionally, it sought to evaluate the occurrence of obesity and alterations in blood glucose and blood lipids following GAHT administration. Furthermore, alterations in lean body mass were assessed.

## Methods

### Study population

**Fig. 1:**
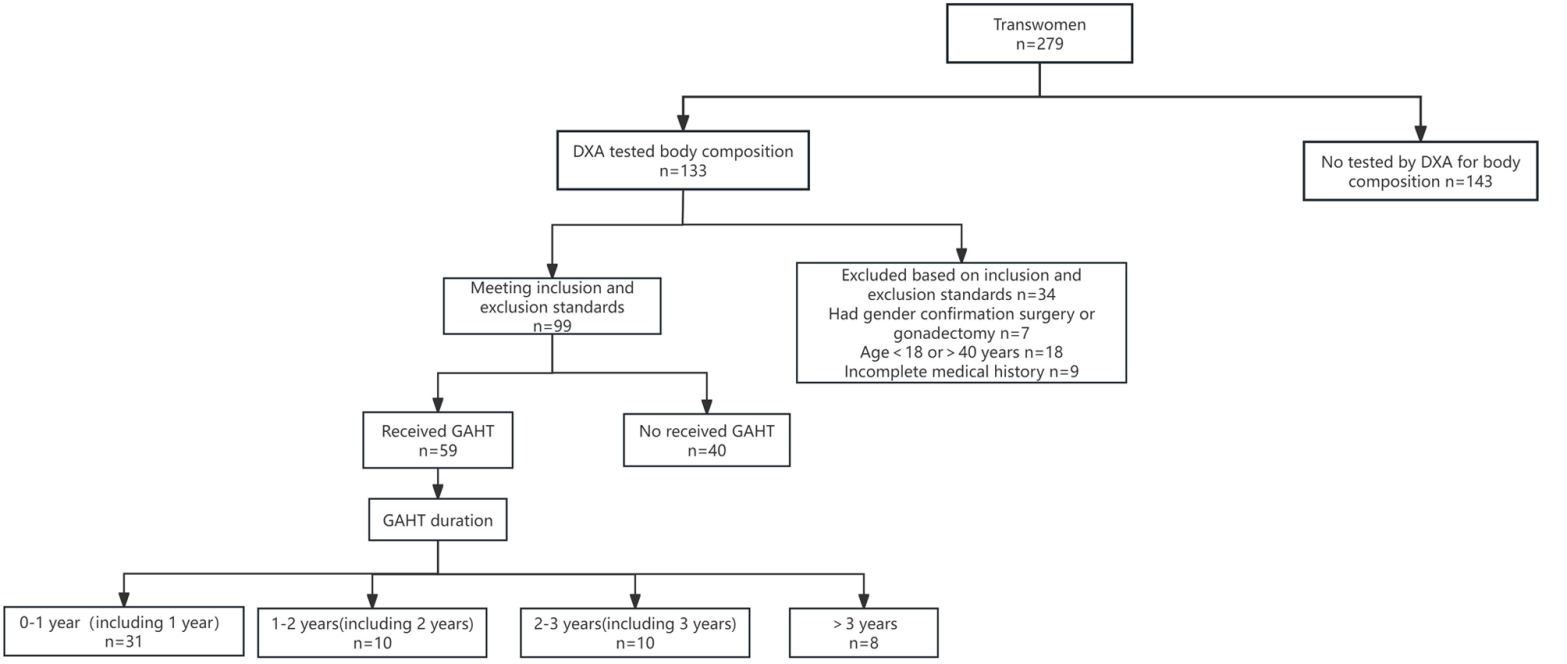
Participant inclusion GAHT: Gender-affirming hormone therapy DXA: Dual energy X-ray absorptiometry

The study subjects were patients in the reproductive endocrinology outpatient department of a tertiary hospital from January 2023 to August 2023, with a total of 279 transwomen. Among them, 133 patients had previously undergone dual energy X-ray absorptiometry (DXA) to assess body composition. Among 99 patients who met the inclusion and exclusion criteria, 40 patients had not received GAHT, and 59 patients had received GAHT. A total of 86 patients had complete blood lipid test results. Lipid data were missing for thirteen patients. Among them, two patients did not receive GAHT, eight patients received GAHT for less than one year, and three patients received GAHT for two years, more than two years (less than three years), and more than three years, respectively. In this study, a direct exclusion method was used to directly exclude missing lipid data. Only well-documented lipid data were statistically analyzed. Eleven patients had missing glycemic data. Of these, two patients did not receive GAHT, eight patients received GAHT for less than one year, and one patient received GAHT for more than two years (less than three years). A direct exclusion method was also used for missing glycemic data. The flow chart depicting the inclusion of participants is illustrated in Fig. 1.

The inclusion criteria for patients were as follows: (1) diagnosed with “transgender female” or “gender identity disorder” or an equivalent diagnosis by the psychiatric department of a top-tier hospital in China and referred to the reproductive endocrinology outpatient clinic with a need for GAHT; (2) body composition testing undergoing DXA; (3) aged between 18 and 40 years, including 18 and 40 years. The exclusion criteria were as follows: (1) unclear duration of GAHT treatment; (2) incomplete medical records; (3) had already undergone GCS or gonadectomy.

### Anthropometric data

Basic demographic information, body composition data, and lipid profile data were gathered from transwomen and classified according to whether they had undergone GAHT. Subjects who had received GAHT were further categorized based on the duration of their hormone therapy. Basic information on demographics was gathered, including age, height, weight, body mass index (BMI), and duration of GAHT. Body composition data included fat content and percentage in each region of the body as well as lean body mass content and percentage in each region of the body. The lipid panel included total cholesterol (TC), high-density lipoprotein-cholesterol (HDL-C), triglyceride (TG), and low-density lipoprotein-cholesterol (LDL-C) data. In addition, this study also collected data on patients’ blood glucose levels.

### GAHT programs

The hormones used for GAHT in this study were mainly estrogens and antiandrogens, with estrogens being mainly estradiol valerate tablets (2–6 mg/day) or estradiol gel (1.5-3 mg/day) and with antiandrogens being mainly cyproterone acetate tablets (6.25-25 mg/day). Estradiol valerate tablets and cyproterone acetate tablets were administered orally, and estradiol gel was administered transdermally. In addition, of the included patients, 6 patients had taken spironolactone tablets (oral administration), 2 patients had used estradiol valerate injection (intramuscular injection), and 1 patient had used estradiol progesterone combination injection (intramuscular injection). Five patients had taken psychotropic medications such as lorazepam tablets and escitalopram oxalate tablets due to psychological problems.

In this study, patients were stratified according to different durations they had been treated with GAHT: 0 represented not treated with GAHT; 0–1 represented treatment duration within one year (including one year); 1–2 represented treatment duration between one and two years (including two years); 2–3 represented treatment duration between two and three years (involving three years); and > 3 represented treatment duration over three years. Patients treated with GAHT are required to follow up every 6 months and undergo medical check-ups, such as liver and kidney function tests, breast ultrasound, and bone densitometry.

### Body composition

Body composition data were calculated utilizing DXA technology using a Lunar Prodigy (GE Healthcare, Chicago, IL, USA). EnCORE software (version 16) was used to analyze all of the chosen data. The region boundaries are generated mechanically by Prodigy enCORE software. The criteria for delineating the various areas of the body are detailed in the supplementary material [15–18]. The percentage of total body fat is the ratio of total body fat content to body weight, and the percentage of fat in each region represents the ratio of fat content in each region to the total fat content in that region. The corrected leg fat content is the fat content of the leg region minus the fat content of the gynoid region.

### Lipid profile tests and blood glucose tests

Blood samples were analyzed for lipid profiles and blood glucose levels using a fully automated biochemical analyzer (Hitachi Model 7180, Japan).

### Diagnostic criteria for obesity

According to the data summarized and analyzed by the Chinese Obesity Task Force, the definition of obesity in adults in China is as follows: individuals with a BMI ≥ 28 are considered obese [19]. According to the recommendations of the American Society of Bariatric Physicians (ASBP) and widely reported criteria for determining obesity in Chinese studies, individuals with a total body fat percentage (TBF%) ≥ 25% for males and ≥ 30% for females are also classified as obese [20].

### Statistical analyses

A database was constructed using Excel software, and SPSS software was used to perform the statistical analysis. All tests were two-tailed, and the significance threshold was set at 0.05. The variable results were normally distributed, so logarithmic transformation was not required before the analysis. The patients’ basic characteristics were assessed using descriptive analysis. The patients were stratified based on the duration of GAHT, and the number of patients in each stratum was represented by the number of case. Body composition data were presented as the mean ± standard deviations, and t tests and analysis of variance were used for single-factor analysis. In cases of heterogeneity of variance among groups, Welch’s ANOVA was used, and in multiple comparisons, the Bonferroni test was used for homogeneous variance, while the Games-Howell test was used for heteroscedasticity. The analysis of count data was performed using the chi-square test.

## Results

### Analysis of the basic situation

The study included 99 transwomen with required demographic and clinical information. Among them, 40 transwomen did not receive GAHT, and 59 transwomen received GAHT. After statistical analysis, there was no statistically significant difference between the two groups in terms of age (25 ± 5 vs. 23 ± 3 years), body height (173.4 ± 5.6 vs. 170.9 ± 6.4 cm), body weight (60.77 ± 8.61 vs. 61.88 ± 10.70 kg), or BMI (20.24 ± 2.92 vs. 21.16 ± 3.22 kg/m^2^), which indicated that the two groups were comparable. The following specific data were analyzed as shown in Tables 1 and 2.

**Table 1:** Basic information on the transwomen

	Categorization		*t*	*P*
	Not received GAHT (*n* = 40)	Received GAHT (*n* = 59)		
Age (years)	25 ± 5	23 ± 3	1.9	0.064
body height (cm)	173.4 ± 5.6	170.9 ± 6.4	2.0	0.05
Body weight (kg)	60.77 ± 8.61	61.88 ± 10.70	-0.5	0.587
Body mass index (kg/m^2^)	20.24 ± 2.92	21.16 ± 3.22	-1.4	0.154

Values are given as the mean ± SD; GAHT: Gender-affirming hormone therapy

**Table 2:** Basic information of transwomen after stratification according to GAHT duration

The duration of treatment	Stratification					*F*	*P*
	0 (*n* = 40)	0–1 (*n* = 31)	1–2 (*n* = 10)	2–3 (*n* = 10)	>3 (*n* = 8)		
Age (years)	25 ± 5	22 ± 3	22 ± 2	24 ± 4	25 ± 4	**–**	0.078
Body height (cm)	173.4 ± 5.6	169.5 ± 7.1	171.5 ± 5.7	173.8 ± 4.1	172.1 ± 5.8	2.1	0.085
Body weight (kg)	60.77 ± 8.61	59.84 ± 11.72	64.67 ± 7.21	65.44 ± 9.12	61.84 ± 11.80	0.9	0.452
Body mass index (kg/m^2^)	20.24 ± 2.92	20.80 ± 3.57	22.02 ± 2.39	21.62 ± 2.54	20.85 ± 3.70	0.9	0.484

Values are given as the mean ± SD; GAHT: Gender-affirming hormone therapy; 0: Not received GAHT; 0–1: 0 < Duration of treatment ≤ 1 year; 1–2: 1 year < Duration of treatment ≤ 2 years; 2–3: 2 years < Duration of treatment ≤ 3 years; >3: Duration of treatment > 3 years

### Effect of GAHT on fat

#### Changes in total body and regional fat content and percentage

Received GAHT compared to not receiving GAHT, the total body fat content increased by 19.65% (*P =* 0.02), and the percentage of total body fat increased by 17.63% (*P =* 0.004). Among the regionalized fat content and percentage, the content and percentage in the arm, leg, corrected leg, and gynoid region increased, with the corrected leg region having the greatest increase in fat content (+ 50.69%, *P* < 0.001) and percentage (+ 34.90%, *P* < 0.001). Visceral fat content decreased by 37.49% (*P =* 0.008). The specific data are provided in Table 3.

**Table 3:** Body fat in transwomen

	Categorization		Magnitude of change	*t*	*P*
	Not received GAHT (*n* = 40)	Received GAHT (*n* = 59)			
**Body fat content**					
Total body (kg)	13.95 ± 5.55	16.69 ± 5.72	19.65%	-2.4	0.020
Arm region (kg)	1.48 ± 0.57	1.83 ± 0.62	24.02%	-2.9	0.005
Leg region (kg)	4.32 ± 1.50	6.11 ± 2.08	41.47%	-4.7	< 0.001
Gynoid region (kg)	2.20 ± 0.86	2.92 ± 0.98	32.60%	-3.7	< 0.001
Corrected leg region(kg)	2.12 ± 0.70	3.19 ± 1.15	50.69%	-5.8	< 0.001
Android region/ Gynoid region	0.44 ± 0.15	0.36 ± 0.08	-18.30%	3.1	0.003
Visceral region(kg)	0.47 ± 0.37	0.29 ± 0.20	-37.49%	2.8	0.008
**Body fat content percentage**					
Total body	0.22 ± 0.07	0.26 ± 0.05	17.63%	-3.0	0.004
Arm region	0.2142 ± 0.07	0.2682 ± 0.06	25.19%	-4.2	< 0.001
Leg region	0.2051 ± 0.06	0.2674 ± 0.05	30.39%	-5.5	< 0.001
Gynoid region	0.23 ± 0.07	0.29 ± 0.06	26.79%	-4.8	< 0.001
Corrected leg region	0.18 ± 0.05	0.25 ± 0.05	34.90%	-5.9	< 0.001
Android region/ Gynoid region	1.03 ± 0.24	0.90 ± 0.14	-12.75%	3.1	0.003

Values are given as the mean ± SD; GAHT: Gender-affirming hormone therapy

### Changes in fat after receiving different durations of GAHT

Receiving GAHT for different durations compared to not receiving GAHT, the fat content and percentage of the arm, leg, corrected leg, gynoid region, and total body, as well as the percentage of fat in the trunk region, showed different degrees of increase. When the groups receiving GAHT of different durations were compared, the maximum impact of fat gain in the total body and different regions of the body was achieved after one to two years of GAHT; however, the maximum impact of visceral fat loss was also achieved after one to two years of GAHT. Multiple comparisons revealed significant difference in the fat content and percentage of the arm, leg, corrected leg, and gynoid region between those who received GAHT for one to two years and those who did not. There were no discrepancies in fat content or percentage of fat in the whole body or body regions (except for the percentage of fat in the arm, the gynoid region, or the total body region) between those who received GAHT within one year, one to two years, two to three years, and more than three years. The detailed information is included in Table 3 of the supplementary material and Table 4.

### Effect of GAHT on the incidence of obesity

Received GAHT compared to not receiving GAHT, the incidence of obesity among transwomen did not alter in a statistically significant way. With a BMI ≥ 28 as the criterion for obesity, the obesity rates of transwomen who did and did not receive GAHT were 1.69% and 0%, respectively. Transwomen who did not receive GAHT had an obesity rate of 45% according to the obesity criterion of TBF% ≥ 25%, and transwomen who received GAHT had an obesity rate of 30.51% according to the obesity criterion of TBF% ≥ 30%. The specific data are provided in Table 5.

**Table 4:** Body fat in transwomen after stratification according to GAHT duration

The duration of treatment	Stratification					*F*	*P*
	0 (*n* = 40)	0–1 (*n* = 31)	1–2 (*n* = 10)	2–3 (*n* = 10)	>3 (*n* = 8)		
**Body fat content**							
Total body (kg)	13.95 ± 5.55	15.20 ± 5.87	18.89 ± 3.15	17.64 ± 5.99	18.52 ± 6.57	2.6	0.038
Arm region (kg)	1.48 ± 0.57	1.65 ± 0.63	2.12 ± 0.39	1.93 ± 0.61	2.09 ± 0.68	4.0	0.005
Leg region (kg)	4.32 ± 1.50	5.48 ± 2.15	7.09 ± 1.38	6.56 ± 2.14	6.75 ± 1.92	7.9	< 0.001
Gynoid region (kg)	2.20 ± 0.86	2.68 ± 1.07	3.30 ± 0.51	3.09 ± 1.09	3.13 ± 0.86	4.7	0.002
Corrected leg region(kg)	2.76 ± 1.12	2.12 ± 0.70	2.80 ± 1.14	3.79 ± 0.90	3.63 ± 1.08	10.6	<0.001
Android region/ Gynoid region	0.44 ± 0.15	0.35 ± 0.06	0.37 ± 0.07	0.36 ± 0.10	0.39 ± 0.13	**–**	0.048
**Body fat content percentage**							
Total body	0.22 ± 0.07	0.25 ± 0.05	0.29 ± 0.03	0.26 ± 0.06	0.29 ± 0.06	**–**	0.001
Arm region	0.21 ± 0.07	0.25 ± 0.06	0.29 ± 0.03	0.27 ± 0.06	0.31 ± 0.05	**–**	< 0.001
Leg region	0.21 ± 0.06	0.25 ± 0.05	0.30 ± 0.04	0.27 ± 0.05	0.29 ± 0.04	10.2	< 0.001
Trunk region	0.25 ± 0.09	0.26 ± 0.07	0.30 ± 0.03	0.27 ± 0.07	0.30 ± 0.08	**–**	0.03
Gynoid region	0.23 ± 0.07	0.28 ± 0.06	0.32 ± 0.03	0.29 ± 0.06	0.32 ± 0.04	**–**	< 0.001
Corrected leg region	0.18 ± 0.05	0.23 ± 0.05	0.28 ± 0.05	0.26 ± 0.05	0.27 ± 0.05	12.4	< 0.001

Values are given as the mean ± SD; GAHT: Gender-affirming hormone therapy; 0: Not received GAHT; 0–1: 0 < Duration of treatment ≤ 1 year; 1–2: 1 year < Duration of treatment ≤ 2 years; 2–3: 2 years < Duration of treatment ≤ 3 years; >3: Duration of treatment > 3 years

**Table 5:** Prevalence of obesity among transwomen not receiving and receiving GAHT

	Categorization		*χ* ^2^	*P*
	Not received GAHT (*n* = 40)	Received GAHT (*n* = 59)		
Body mass index ≥ 28	0	1	<0.01	1
TBF% ≥ 25%(Not received GAHT), TBF% ≥ 30%(Received GAHT)	18	18	2.2	0.141

Values are given as the mean ± SD; GAHT: Gender-affirming hormone therapy; TBF%: Total body fat percentage

### Effect of GAHT on blood lipid levels and blood glucose levels

Received GAHT compared to not receiving GAHT, the TC in the body decreased (-10.45%, *P =* 0.011), while there were no meaningful alterations in TG, LDL-C, or HDL-C. The levels of four blood lipids before and after treatment were within the normal range. The specific data are provided in Table 6.

**Table 6:** Lipids in transwomen not receiving and receiving GAHT

	Categorization		Magnitude of change	*t*	*P*	Normal value reference range
	Not received GAHT (*n* = 38)	Received GAHT (*n* = 48)				
Total cholesterol(mmol/L)	4.81 ± 1.02	4.31 ± 0.67	-10.45%	2.6	0.011	3.1–5.7
Triglycerides(mmol/L)	1.05 ± 0.55	0.95 ± 0.41	-9.45%	1.0	0.338	0.56–1.7
High-Density Lipoprotein-cholesterol(mmol/L)	1.28 ± 0.30	1.31 ± 0.25	2.43%	-0.5	0.597	0.91–2.05
Low-Density Lipoprotein-cholesterol(mmol/L)	2.73 ± 0.73	2.46 ± 0.54	-9.69%	1.9	0.057	1.57–3.76

Values are given as the mean ± SD; GAHT: Gender-affirming hormone therapy

Received GAHT compared to not receiving GAHT, body glucose levels were elevated in the GAHT-treated group (+ 12.31%, *P* < 0.001). The specific data are provided in Table 7. When groups receiving GAHT for different durations were compared, blood glucose levels continued to increase with increasing time on GAHT (*P* = 0.004). The specific data are provided in Table 8.

**Table 7:** Blood glucose levels in transwomen not receiving and receiving GAHT

	Categorization		Magnitude of change	*t*	*P*	Normal value reference range
	Not received GAHT (*n* = 38)	Received GAHT (*n* = 50)				
Blood glucose(mmol/L)	5.03 ± 0.64	5.65 ± 0.79	12.31%	4.0	<0.001	3.89–6.11

Values are given as the mean ± SD; GAHT: Gender-affirming hormone therapy

**Table 8:** Blood glucose levels in transwomen after stratification according to GAHT duration

	Stratification					*F*	*P*
	0 (*n* = 38)	0–1 (*n* = 22)	1–2 (*n* = 10)	2–3 (*n* = 9)	>3 (*n* = 8)		
Blood glucose(mmol/L)	5.03 ± 0.64	5.25 ± 0.40	5.52 ± 0.37	6.15 ± 0.97	6.22 ± 0.79	**–**	0.004

Values are given as the mean ± SD; GAHT: Gender-affirming hormone therapy; 0: Not received GAHT; 0–1: 0 < Duration of treatment ≤ 1 year; 1–2: 1 year < Duration of treatment ≤ 2 years; 2–3: 2 years < Duration of treatment ≤ 3 years; >3: Duration of treatment > 3 years

### Effect of GAHT on lean body mass

#### Changes in total body and regional lean body mass content and percentage

Received GAHT compared to not receiving GAHT, the percentage of total lean body mass (-5.13%, *P =* 0.004) was lower in transwomen. The lean body mass content and percentage of the arm region decreased by -8.65% (*P* = 0.005) and − 7.59% (*P* < 0.001), respectively. Furthermore, the percentages of lean body mass in the leg, corrected leg, and gynoid regions decreased by -7.59% (*P* < 0.001), -6.43% (*P* < 0.001) and − 7.79% (*P* < 0.001), respectively. The specific data are provided in Table 1 of the supplementary material.

### Changes in lean body mass after receiving different durations of GAHT

Receiving GAHT for different durations compared to not receiving GAHT, the content and percentage of lean body mass in the arm region and the percentage of lean body mass in the leg, corrected leg, trunk, gynoid, and total body regions decreased to variable degrees. The results of multiple comparisons mostly showed significant differences in the lean body mass percentage of the total body, arm region, leg region, corrected leg region, trunk region, and gynoid region between those who received GAHT for one to two years and those who did not. Those who received GAHT within one year and those who received GAHT for one to two years showed notable variations in the percentage of lean body mass in the arm, gynoid region, and total body. There were no discernible discrepancies in the content of lean body mass or the percentage of lean body mass in different regions (except for the arm region and gynoid region) among those who underwent GAHT within one year, one to two years, two to three years, or more than three years. The specific data are provided in Tables 2 and 3 of the supplementary material.

## Discussion

A retrospective analysis was conducted on body fat and lipid data from transwomen who did and did not receive GAHT. The aims of this research were to figure out the effects of GAHT on body fat and lipid profiles in transwomen and to analyze the alterations in body fat associated with different durations of treatment. These findings may contribute to understanding the process of fat redistribution in Chinese transwomen after receiving GAHT and the potential increased risk of CVD.

This study revealed that after receiving GAHT, transwomen experience increases in both total and regional body fat, with the corrected leg region showing the most significant increase, followed by the leg region. Additionally, the fat of the gynoid region also increases, and the fat ratio between the android and gynoid regions decreases. Previous research has indicated that GAHT may have an impact on body fat through both direct and indirect mechanisms. The direct mechanism involves the binding of estrogen to estrogen receptors, which promotes the body’s storage of fat by stimulating the growth of preadipocytes and lipoprotein lipase activity. The indirect mechanism involves the suppression of pituitary gonadotropin secretion through anti-androgen effects, leading to a decrease in testosterone levels. Lipoprotein lipase activity is inhibited, and lipolysis in adipose tissue is increased when testosterone binds to androgen receptors. As a result of these two mechanisms, body fat changes and is redistributed after GAHT in transwomen. These changes are similar to those observed during puberty in cisgender females, where increased estrogen levels lead to fat primarily being stored in the hip and leg region, thereby forming the typical female body shape [21]. Additionally, Klaver et al. [5] reported a considerable increase in fat of the leg and gynoid region after one year of GAHT treatment in transwomen. Therefore, after receiving GAHT, fat begins to redistribute and accumulate in a more feminized manner, resulting in a gradual transition from a masculine to a feminine body shape.

Although it is possible that having more body fat will increase one’s risk of obesity, the incidence of obesity did not change significantly in this study. It is usually assumed that changes in body fat trigger changes in blood lipids. Velzen et al. [13] reported that after one year of treatment with estrogen plus cyproterone acetate, the levels of all four blood lipids decreased to varying degrees. Similarly, Cocchetti et al. [22] reported significant decreases in TG, TC, and LDL-C in transwomen receiving GAHT after a two-year follow-up. The present study further explored alterations in lipids in transwomen who had received GAHT after observing body fat redistribution. This study revealed that LDL-C, TG, and TC levels tended to decrease in Chinese transwomen after hormone administration, with the most significant decrease in TC levels. Importantly, the lipid levels after receiving GAHT were all within the normal range, which to some extent indicated that the increase in body fat in transwomen was within a reasonable range. In addition, this study revealed a significant decrease in visceral fat content after receiving GAHT. However, exogenous estradiol is linked to changes in glucose metabolism [23]. Exogenous estradiol might prevent the binding of insulin to its receptor, which could lead to body resistance to the effects of insulin and cause elevated blood glucose [24]. Thus, although none of the changes in obesity, visceral fat, or lipids suggested an increased risk of cardiovascular occurrence, the elevated blood glucose levels prompted us to focus on how estrogen causes changes in blood glucose in subsequent studies and whether such changes exacerbate the risk of CVD in transwomen [25–27].

The present study revealed a significant reduction in visceral fat content after receiving GAHT. However, Klaver Maartje et al. [28] reported only minor changes in visceral adiposity within one year before and after GAHT. This difference might be due to the varying duration of GAHT in the present study, which ranged from less than one year to more than three years. Therefore, it might take a long treatment period to observe significant changes in visceral fat content [29].

In addition, the fat content percentage of each region (Arm, Leg, Trunk, Android, Gynoid, and Corrected leg) in transwomen who had received GAHT was between that of ciswomen and cismen, as reported in previous studies [15, 30–33]. This is in comparison to previous studies of cisgender males and females. According to the Fifth National Physical Fitness Monitoring Bulletin issued by the National Center for National Physical Fitness Monitoring [34], TBF% of transwomen who received GAHT in this study fell within the range of TBF% of cisgender females aged 20–39 years (24.9—29.1%). These results imply that the increase in body fat of transwomen is a desirable outcome and that fat accumulation and distribution in various regions after receiving GAHT move toward a more feminine body shape.

In previous studies, the duration of GAHT interventions has typically been 1 year. Some studies have hypothesized that GAHT has the greatest effect on changes in body composition 2–5 years after initiation [35]. However, no randomized controlled trials have consistently observed changes in body composition 2–5 years after GAHT. In the present study, most of the multiple comparisons showed statistically significant differences in body fat status between patients who had received GAHT for one to two years and those who had not. Therefore, it is hypothesized that the shift from masculinized to feminized body fat is estimated to reach a homeostatic state in one to two years in transwomen after treatment with GAHT, especially in the leg and gynoid region.

### Strengths and limitations

This study’s primary strength is that it provides an in-depth study of transwomen in China, a social group that has previously received inadequate attention. Focusing on the impact of GAHT on changes in fat in various parts of the body, this study comprehensively investigated whether increased fat may increase the risk of developing CVD [36], including comprehensive consideration of key indicators such as visceral fat content and the prevalence of obesity, glucose, and blood lipids. However, this was a case‒control study and not a before‒and‒after control study, so the conclusions obtained have some limitations. Moreover, patients’ lifestyles, different hormone therapy regimens, and incomplete lipid data can all have an impact on the study results. This study suggests that a randomized controlled study using one uniform GAHT protocol be conducted for future research to better understand the changes in body composition. In addition, it is recommended that data on participants’ physical appearance and lifestyle, such as circumference and breast development and activity status, be collected during the study period, as well as long-term follow-ups, to obtain a more comprehensive picture of physical changes, such as the extent and timing of changes.

## Conclusion

After receiving GAHT, Chinese transwomen exhibited increases in their total and regional fat content as well as a shift in their body fat distribution from masculine to feminine. Body fat accumulates more in the leg and gynoid region, especially during the first two years. However, the visceral fat content decreased, and the increase in TBF% did not lead to significant changes in the incidence of obesity or TG or LDL-C. In addition, blood glucose levels were elevated, suggesting that future studies could focus on whether elevated blood glucose from GAHT increases the risk of CVD. Fat distribution and accumulation tended to stabilize after 1–2 years of GAHT treatment. The results of this study can help clinicians better understand the changes in the body composition of transwomen after receiving GAHT to develop a more precise hormone therapy program for patients. In addition, this study revealed the effects of GAHT on key indicators, such as visceral fat content, incidence of obesity, blood glucose, and blood lipids, which can help clinicians adjust treatment programs promptly and prevent the occurrence of CVD in patients by monitoring changes in these indicators during the treatment process.

### Electronic supplementary material

Below is the link to the electronic supplementary material.

Supplementary Material 1

Supplementary Material 2

Supplementary Material 3

## Data Availability

The corresponding author will make the datasets used and analyzed during the study available upon reasonable request.

## Abbreviations

GAHT: Gender-affirming hormone therapy
GCS: Gender confirmation surgery
CVD: Cardiovascular disease
DXA: Dual energy X-ray absorptiometry
BMI: Body mass index
TC: Total cholesterol
HDL-C: High-density lipoprotein-cholesterol
TG: Triglyceride
LDL-C: Low-density lipoprotein-cholesterol
ASBP: American Society of Bariatric Physicians
TBF%: Total body fat percentage
