# Supplementary material for: Effects of gender-affirming hormone therapy on body fat: a retrospective case‒control study in Chinese transwomen
Source: Lipids Health Dis. 2024 May 17;23:146. doi: 10.1186/s12944-024-02131-y (PMC11100057; doi:10.1186/s12944-024-02131-y)
Supplement: Supplementary file 1 — Supplementary Material 1 [file 12944_2024_2131_MOESM1_ESM.docx]

**Supplementary materia**

**Table1-3:Values are given as mean ± SD; GAHT：Gender-Affirming Hormone Therapy**

**Table 1 Body fat and Lean body mass in transwomen**

|  | **Categorization** | | **magnitude of change** | ***t*** | ***P*** |
| --- | --- | --- | --- | --- | --- |
|  | **Not received GAHT (n = 40)** | **Received GAHT**  **(n = 59)** |  |  |  |
| Body Fat Content |  |  |  |  |  |
| Total body (g) | 13947.84 ± 5548.74 | 16688.01 ± 5723.39 | 19.65% | -2.366 | 0.02 |
| Arm region (g) | 1479.29 ± 566.17 | 1834.63 ± 618.96 | 24.02% | -2.9 | 0.005 |
| Leg region (g) | 4317.83 ± 1495.52 | 6108.64 ± 2076.82 | 41.47% | -4.688 | <.001 |
| Trunk region (g) | 7183.31 ± 3609.06 | 7786.01 ± 3144.92 | 8.39% | -0.881 | 0.38 |
| Android region (g) | 1046.55 ± 652.23 | 1097.72 ± 568.78 | 4.89% | -0.414 | 0.68 |
| Gynoid region (g) | 2199.96 ± 861.76 | 2917.25 ± 984.02 | 32.60% | -3.738 | <.001 |
| Android region/ Gynoid region | 0.44 ± 0.15 | 0.36 ± 0.08 | -18.30% | 3.141 | 0.003 |
| Visceral region | 468.10 ± 365.49 | 292.61 ± 203.47 | -37.49% | 2.761 | 0.008 |
| Body Fat Content Percentage |  |  |  |  |  |
| Total body | 0.22 ± 0.07 | 0.26 ± 0.05 | 17.63% | -3.023 | 0.004 |
| Arm region | 0.2142 ± 0.07 | 0.2682 ± 0.06 | 25.19% | -4.16 | <.001 |
| Leg region | 0.2051 ± 0.06 | 0.2674 ± 0.05 | 30.39% | -5.472 | <.001 |
| Trunk region | 0.25 ± 0.09 | 0.27 ± 0.07 | 10.84% | -1.56 | 0.123 |
| Android region | 0.25 ± 0.11 | 0.27 ± 0.08 | 8.25% | -0.993 | 0.324 |
| Gynoid region | 0.23 ± 0.07 | 0.29 ± 0.06 | 26.79% | -4.759 | <.001 |
| Android region/ Gynoid region | 1.03 ± 0.24 | 0.90 ± 0.14 | -12.75% | 3.074 | 0.003 |
| Body Fat Content Distribution |  |  |  |  |  |
| Arm region | 0.11 ± 0.01 | 0.11 ± 0.01 | 3.12% | -1.462 | 0.147 |
| Leg region | 0.32 ± 0.04 | 0.37 ± 0.03 | 15.02% | -6.347 | <.001 |
| Trunk region | 0.49 ± 0.07 | 0.46 ± 0.04 | -6.91% | 2.859 | 0.006 |
| Android region | 0.07 ± 0.02 | 0.06 ± 0.01 | -8.72% | 1.8 | 0.077 |
| Gynoid region | 0.16 ± 0.02 | 0.18 ± 0.01 | 10.67% | -5.937 | <.001 |
| Visceral region | 0.03 ± 0.01 | 0.02 ± 0.01 | -44.67% | 5.971 | <.001 |
| Lean Body Mass Content |  |  |  |  |  |
| Total body (g) | 46825.97 ± 5664.00 | 45191.46 ± 6158.96 | -3.49% | 1.338 | 0.184 |
| Arm region (g) | 5342.27 ± 885.48 | 4880.39 ± 708.26 | -8.65% | 2.875 | 0.005 |
| Leg region (g) | 16541.77 ± 2398.13 | 16268.07 ± 2631.16 | -1.65% | 0.526 | 0.6 |
| Trunk region (g) | 20841.53 ± 2443.50 | 20008.60 ± 2879.61 | -4.00% | 1.499 | 0.137 |
| Android region (g) | 2907.96 ± 347.00 | 2819.36 ± 443.59 | -3.05% | 1.062 | 0.291 |
| Gynoid region (g) | 7116.42 ± 964.59 | 6849.21 ± 1088.32 | -3.75% | 1.254 | 0.213 |
| Android region/ Gynoid region | 0.41 ± 0.02 | 0.41 ± 0.03 | 0.00% | -0.513 | 0.609 |
| Lean Body Mass Content Percentage |  |  |  |  |  |
| Total body | 0.78 ± 0.07 | 0.74 ± 0.05 | -5.13% | 3.023 | 0.004 |
| Arm region | 0.79 ± 0.07 | 0.73 ± 0.06 | -7.59% | 4.16 | <.001 |
| Leg region | 0.79 ± 0.06 | 0.73 ± 0.05 | -7.59% | 5.472 | <.001 |
| Trunk region | 0.75 ± 0.09 | 0.73 ± 0.07 | -2.67% | 1.56 | 0.123 |
| Android region | 0.75 ± 0.11 | 0.73 ± 0.08 | -2.67% | 0.993 | 0.324 |
| Gynoid region | 0.77 ± 0.07 | 0.71 ± 0.06 | -7.79% | 4.759 | <.001 |
| Android region/ Gynoid region | 0.98 ± 0.08 | 1.04 ± 0.06 | 6.12% | -4.274 | <.001 |
| Lean Body Mass Content Distribution |  |  |  |  |  |
| Arm region | 0.1137 ± 0.01 | 0.1081 ± 0.01 | -4.93% | 3.275 | 0.001 |
| Leg region | 0.35 ± 0.01 | 0.36 ± 0.02 | 2.86% | -2.086 | 0.04 |
| Trunk region | 0.45 ± 0.01 | 0.44 ± 0.02 | -2.22% | 0.992 | 0.323 |
| Android region | 0.06 ± 0.00 | 0.06 ± 0.00 | 0.00% | -0.166 | 0.869 |
| Gynoid region | 0.15 ± 0.01 | 0.15 ± 0.01 | 0.00% | 0.364 | 0.717 |

**Table 2 Body fat and Lean body mass in tranwomen after stratification according to GAHT duration**

| The duration of treatment | **Stratification** | | | | | ***F*** | ***P*** | **Reference value cis women** | **Reference value cis men** |
| --- | --- | --- | --- | --- | --- | --- | --- | --- | --- |
|  | **0**  **(n = 40)** | **0-1**  **(n = 31)** | **1 -2**  **(n = 10)** | **2-3**  **(n = 10)** | **＞3**  **(n = 8)** |  |  |  |  |
| Body Fat Content |  |  |  |  |  |  |  |  |  |
| Total body (g) | 13947.84 ± 5548.74 | 15198.84 ± 5865.70 | 18887.15 ± 3154.65 | 17636.49 ± 5988.66 | 18523.98 ± 6571.68 | 2.647 | 0.038 |  |  |
| Arm region (g) | 1479.29 ± 566.17 | 1646.40 ± 626.15 | 2120.69 ± 387.54 | 1928.32 ± 607.01 | 2089.31 ± 683.23 | 4.038 | 0.005 |  |  |
| Leg region (g) | 4317.83 ± 1495.52 | 5479.87 ± 2151.41 | 7091.80 ± 1378.89 | 6559.14 ± 2139.18 | 6753.06 ± 1917.49 | 7.86 | <.001 |  |  |
| Trunk region (g) | 7183.31 ± 3609.06 | 7110.61 ± 3156.50 | 8716.34 ± 1711.77 | 8171.37 ± 3438.05 | 8758.54 ± 3966.74 | 0.907 | 0.463 |  |  |
| Android region (g) | 1046.55 ± 652.23 | 984.42 ± 541.73 | 1217.39 ± 318.87 | 1174.70 ± 685.85 | 1290.99 ± 747.52 | 0.656 | 0.624 |  |  |
| Gynoid region (g) | 2199.96 ± 861.76 | 2682.06 ± 1066.95 | 3301.54 ± 510.32 | 3093.67 ± 1085.40 | 3127.69 ± 862.36 | 4.662 | 0.002 |  |  |
| Android region/ Gynoid region | 0.44 ± 0.15 | 0.35 ± 0.06 | 0.37 ± 0.07 | 0.36 ± 0.10 | 0.39 ± 0.13 |  | 0.048 |  |  |
| Visceral region | 468.10 ± 365.49 | 280.57 ± 213.23 | 246.00 ± 119.21 | 321.43 ± 172.97 | 361.51 ± 284.72 |  | 0.057 |  |  |
| Body Fat Content Percentage |  |  |  |  |  |  |  |  |  |
| Total body | 0.22 ± 0.07 | 0.25 ± 0.05 | 0.29 ± 0.03 | 0.26 ± 0.06 | 0.29 ± 0.06 |  | 0.001 |  |  |
| Arm region | 0.21 ± 0.07 | 0.25 ± 0.06 | 0.29 ± 0.03 | 0.27 ± 0.06 | 0.31 ± 0.05 |  | <.001 | 0.31^26^ | 0.14^26^ |
| Leg region | 0.21 ± 0.06 | 0.25 ± 0.05 | 0.30 ± 0.04 | 0.27 ± 0.05 | 0.29 ± 0.04 | 10.202 | <.001 | 0.36^26^ | 0.18^26^ |
| Trunk region | 0.25 ± 0.09 | 0.26 ± 0.07 | 0.30 ± 0.03 | 0.27 ± 0.07 | 0.30 ± 0.08 |  | 0.03 | 0.36^27^ | 0.18^29^ |
| Android region | 0.25 ± 0.11 | 0.25 ± 0.08 | 0.30 ± 0.05 | 0.26 ± 0.09 | 0.31 ± 0.10 |  | 0.113 | 0.35^28^ | 0.25^13^ |
| Gynoid region | 0.23 ± 0.07 | 0.28 ± 0.06 | 0.32 ± 0.03 | 0.29 ± 0.06 | 0.32 ± 0.04 |  | <.001 | 0.42^28^ | 0.24^13^ |
| Android region/ Gynoid region | 1.03 ± 0.24 | 0.89 ± 0.12 | 0.92 ± 0.11 | 0.88 ± 0.16 | 0.94 ± 0.23 |  | 0.059 |  |  |
| Body Fat Content Distribution |  |  |  |  |  |  |  |  |  |
| Arm region | 0.11 ± 0.01 | 0.11 ± 0.01 | 0.11 ± 0.01 | 0.11 ± 0.01 | 0.11 ± 0.01 | 0.9 | 0.467 |  |  |
| Leg region | 0.32 ± 0.04 | 0.36 ± 0.03 | 0.37 ± 0.03 | 0.38 ± 0.04 | 0.37 ± 0.03 | 10.547 | <.001 |  |  |
| Trunk region | 0.49 ± 0.07 | 0.46 ± 0.04 | 0.46 ± 0.04 | 0.45 ± 0.05 | 0.46 ± 0.05 |  | 0.145 |  |  |
| Android region | 0.07 ± 0.02 | 0.06 ± 0.01 | 0.06 ± 0.01 | 0.06 ± 0.01 | 0.07 ± 0.01 | 1.043 | 0.389 |  |  |
| Gynoid region | 0.16 ± 0.02 | 0.18 ± 0.01 | 0.18 ± 0.01 | 0.17 ± 0.02 | 0.17 ± 0.02 |  | <.001 |  |  |
| Visceral region | 0.03 ± 0.01 | 0.02 ± 0.01 | 0.01 ± 0.01 | 0.02 ± 0.01 | 0.02 ± 0.01 | 11.213 | <.001 |  |  |
| Lean Body Mass Content |  |  |  |  |  |  |  |  |  |
| Total body (g) | 46825.97 ± 5664.00 | 44641.71 ± 6930.55 | 45784.49 ± 4819.35 | 47800.43 ± 4627.90 | 43319.20 ± 5956.22 | 1.214 | 0.31 |  |  |
| Arm region (g) | 5342.27 ± 885.48 | 4818.17 ± 780.14 | 5052.90 ± 669.37 | 5186.66 ± 444.42 | 4523.01 ± 615.07 | 3.061 | 0.02 |  |  |
| Leg region (g) | 16541.77 ± 2398.13 | 16019.18 ± 3072.42 | 16550.27 ± 1735.83 | 16915.35 ± 1978.57 | 16070.64 ± 2639.60 | 0.342 | 0.849 |  |  |
| Trunk region (g) | 20841.53 ± 2443.50 | 19726.45 ± 3125.86 | 20235.42 ± 2543.93 | 21527.67 ± 2239.66 | 18919.56 ± 2642.53 | 1.805 | 0.134 |  |  |
| Android region (g) | 2907.96 ± 347.00 | 2774.19 ± 490.35 | 2808.76 ± 323.59 | 3078.95 ± 388.05 | 2683.11 ± 380.88 | 1.658 | 0.166 |  |  |
| Gynoid region (g) | 7116.42 ± 964.59 | 6788.60 ± 1234.84 | 6851.75 ± 717.07 | 7251.75 ± 922.46 | 6577.74 ± 1091.72 | 0.922 | 0.455 |  |  |
| Android region/ Gynoid region | 0.41 ± 0.02 | 0.41 ± 0.03 | 0.41 ± 0.02 | 0.43 ± 0.02 | 0.41 ± 0.03 | 0.892 | 0.472 |  |  |
| Lean Body Mass Content Percentage |  |  |  |  |  |  |  |  |  |
| Total body | 0.78 ± 0.07 | 0.75 ± 0.05 | 0.71 ± 0.03 | 0.74 ± 0.06 | 0.71 ± 0.06 |  | 0.001 |  |  |
| Arm region | 0.79 ± 0.07 | 0.75 ± 0.06 | 0.71 ± 0.03 | 0.73 ± 0.06 | 0.69 ± 0.05 |  | <.001 | 0.61^26^ | 0.81^26^ |
| Leg region | 0.79 ± 0.06 | 0.75 ± 0.05 | 0.70 ± 0.04 | 0.73 ± 0.05 | 0.71 ± 0.04 | 10.202 | <.001 | 0.62^26^ | 0.78^26^ |
| Trunk region | 0.75 ± 0.09 | 0.74 ± 0.07 | 0.70 ± 0.03 | 0.73 ± 0.07 | 0.70 ± 0.08 |  | 0.03 | 0.64^27^ | 0.76^29^ |
| Android region | 0.75 ± 0.11 | 0.75 ± 0.08 | 0.70 ± 0.05 | 0.74 ± 0.09 | 0.69 ± 0.10 |  | 0.113 |  |  |
| Gynoid region | 0.77 ± 0.07 | 0.72 ± 0.06 | 0.68 ± 0.03 | 0.71 ± 0.06 | 0.68 ± 0.04 |  | <.001 |  |  |
| Android region/ Gynoid region | 0.98 ± 0.08 | 1.04 ± 0.04 | 1.04 ± 0.05 | 1.04 ± 0.07 | 1.01 ± 0.10 |  | 0.009 |  |  |
| Lean Body Mass Content Distribution |  |  |  |  |  |  |  |  |  |
| Arm region | 0.11 ± 0.01 | 0.11 ± 0.01 | 0.11 ± 0.01 | 0.11 ± 0.01 | 0.10 ± 0.01 | 3.163 | 0.017 |  |  |
| Leg region | 0.35 ± 0.01 | 0.36 ± 0.02 | 0.36 ± 0.01 | 0.35 ± 0.01 | 0.37 ± 0.02 | 2.521 | 0.046 |  |  |
| Trunk region | 0.45 ± 0.01 | 0.44 ± 0.02 | 0.44 ± 0.02 | 0.45 ± 0.01 | 0.44 ± 0.01 | 1.346 | 0.259 |  |  |
| Android region | 0.06 ± 0.00 | 0.06 ± 0.00 | 0.06 ± 0.00 | 0.06 ± 0.00 | 0.06 ± 0.00 |  | 0.101 |  |  |
| Gynoid region | 0.15 ± 0.01 | 0.15 ± 0.01 | 0.15 ± 0.01 | 0.15 ± 0.01 | 0.15 ± 0.01 | 0.165 | 0.956 |  |  |

0: Not received GAHT; 0-1: 0＜Duration of treatment≤1 year; 1-2: 1 year＜Duration of treatment≤2 years; 2-3: 2 years＜Duration of treatment≤3 years; ＞3: Duration of treatment＞3 years

Age categories for reference values; Women: ^26^ category 20-29 years old, ^27^ median (range) 31 (18-62) years old, ^28^ mean (SD) 20.8 (1.9) years old; Men: ^26^ category 20-29 years old, ^29^ median (range) 31 (18-55) years old, ^13^ mean (SD) 21.3 (2.2).

**Table 3 Results of multiple comparisons of body fat and lean body mass in transwomen after stratification according to GAHT duration**

|  | **GAHT duration** | **GAHT duration** | ***P*** | ***95%Cl*** |
| --- | --- | --- | --- | --- |
| Body Fat Content |  |  |  |  |
| Arm region (g) | 0 | 1-2 | 0.026 | (-1236.27,-46.54) |
| Leg region (g) | 0 | 1-2 | 0.000 | (-4620.10,-927.84) |
|  | 0 | 2-3 | 0.007 | (-4087.45,-395.18) |
|  | 0 | ＞3 | 0.008 | (-4457.57,-412.90) |
| Gynoid region (g) | 0 | 1-2 | 0.012 | (-2046.62,-156.53) |
| Android region/ Gynoid region | 0 | 0-1 | 0.009 | (0.02,0.16) |
| Body Fat Mass Percentage |  |  |  |  |
| Total body | 0 | 1-2 | 0.000 | (-0.11,-0.03) |
|  | 0-1 | 1-2 | 0.022 | (-0.08,0) |
| Arm region | 0 | 1-2 | 0.000 | (-0.12,-0.04) |
|  | 0 | ＞3 | 0.005 | (-0.16,-0.03) |
|  | 0-1 | 1-2 | 0.048 | (-0.09,0) |
| Leg region | 0 | 0-1 | 0.011 | (-0.08,-0.01) |
|  | 0 | 1-2 | 0.000 | (-0.15,-0.04) |
|  | 0 | 2-3 | 0.004 | (-0.12,-0.01) |
|  | 0 | ＞3 | 0.001 | (-0.15,-0.03) |
| Trunk region | 0 | 1-2 | 0.032 | (-0.11,0) |
| Gynoid region | 0 | 0-1 | 0.042 | (-0.09,0) |
|  | 0 | 1-2 | 0.000 | (-0.14,-0.05) |
|  | 0 | ＞3 | 0.001 | (-0.14,-0.03) |
|  | 0-1 | 1-2 | 0.030 | (-0.09,0) |
| Body Fat Mass Distribution |  |  |  |  |
| Leg region | 0 | 0-1 | 0.000 | (-0.07,-0.02) |
|  | 0 | 1-2 | 0.000 | (-0.09,-0.02) |
|  | 0 | 2-3 | 0.000 | (-0.09,-0.02) |
|  | 0 | ＞3 | 0.003 | (-0.10,-0.01) |
| Gynoid region | 0 | 0-1 | 0.000 | (-0.03,-0.01) |
|  | 0 | 1-2 | 0.003 | (-0.03,0) |
| Lean Body Mass Percentage |  |  |  |  |
| Total body | 0 | 1-2 | 0.000 | (0.03,0.11) |
|  | 0-1 | 1-2 | 0.022 | (0,0.08) |
| Arm region | 0 | 1-2 | 0.000 | (0.04,0.12) |
|  | 0 | ＞3 | 0.005 | (0.03,0.16) |
|  | 0-1 | 1-2 | 0.048 | (0,0.09) |
| Leg region | 0 | 0-1 | 0.011 | (0.01,0.08) |
|  | 0 | 1-2 | 0.000 | (0.04,0.15) |
|  | 0 | 2-3 | 0.004 | (0.01,0.12) |
|  | 0 | ＞3 | 0.001 | (0.03,0.15) |
| Trunk region | 0 | 1-2 | 0.032 | (0,0.11) |
| Gynoid region | 0 | 0-1 | 0.042 | (0,0.09) |
|  | 0 | 1-2 | 0.000 | (0.05,0.14) |
|  | 0 | ＞3 | 0.001 | (0.03,0.14) |
|  | 0-1 | 1-2 | 0.030 | (0,0.09) |
| Android region/ Gynoid region | 0 | 0-1 | 0.001 | (-0.10,0.02) |

0: Not received GAHT; 0-1: 0＜Duration of treatment≤1 year; 1-2: 1 year＜Duration of treatment≤2 years; 2-3: 2 years＜Duration of treatment≤3 years; ＞3: Duration of treatment＞3 years
