# Supplementary material for: Effects of gender-affirming hormone therapy on body fat: a retrospective case‒control study in Chinese transwomen
Source: Lipids Health Dis. 2024 May 17;23:146. doi: 10.1186/s12944-024-02131-y (PMC11100057; doi:10.1186/s12944-024-02131-y)
Supplement: Supplementary file 2 — Supplementary Material 2 [file 12944_2024_2131_MOESM2_ESM.docx]

**Supplementary materia**

**Table1-3:Values are given as mean ± SD; GAHT：Gender-Affirming Hormone Therapy**

**Table 1 Body fat and Lean body mass in transwomen**

|  | **Categorization** | | **magnitude of change** | ***t*** | ***P*** |
| --- | --- | --- | --- | --- | --- |
|  | **Not received GAHT (n = 40)** | **Received GAHT**  **(n = 59)** |  |  |  |
| Body Fat Content |  |  |  |  |  |
| Total body (kg) | 13.95 ± 5.55 | 16.69 ± 5.72 | 19.65% | -2.4 | 0.02 |
| Arm region (kg) | 1.48 ± 0.57 | 1.83 ± 0.62 | 24.02% | -2.9 | 0.005 |
| Leg region (kg) | 4.32 ± 1.50 | 6.11 ± 2.08 | 41.47% | -4.7 | <0.001 |
| Trunk region (kg) | 7.18 ± 3.61 | 7.79 ± 3.14 | 8.39% | -0.9 | 0.38 |
| Android region (kg) | 1.05 ± 0.65 | 1.10 ± 0.57 | 4.89% | -0.4 | 0.68 |
| Gynoid region (kg) | 2.20 ± 0.86 | 2.92 ± 0.98 | 32.60% | -3.7 | <0.001 |
| Corrected leg region(kg) | 2.12 ± 0.70 | 3.19 ± 1.15 | 50.69% | -5.8 | <0.001 |
| Android region/ Gynoid region | 0.44 ± 0.15 | 0.36 ± 0.08 | -18.30% | 3.1 | 0.003 |
| Visceral region(kg) | 0.47 ± 0.37 | 0.29 ± 0.20 | -37.49% | 2.8 | 0.008 |
| Body Fat Content Percentage |  |  |  |  |  |
| Total body | 0.22 ± 0.07 | 0.26 ± 0.05 | 17.63% | -3.0 | 0.004 |
| Arm region | 0.21 ± 0.07 | 0.27 ± 0.06 | 25.19% | -4.2 | <0.001 |
| Leg region | 0.21 ± 0.06 | 0.27 ± 0.05 | 30.39% | -5.5 | <0.001 |
| Trunk region | 0.25 ± 0.09 | 0.27 ± 0.07 | 10.84% | -1.6 | 0.123 |
| Android region | 0.25 ± 0.11 | 0.27 ± 0.08 | 8.25% | -1.0 | 0.324 |
| Gynoid region | 0.23 ± 0.07 | 0.29 ± 0.06 | 26.79% | -4.8 | <0.001 |
| Corrected leg region | 0.18 ± 0.05 | 0.25 ± 0.05 | 34.90% | -5.9 | <0.001 |
| Android region/ Gynoid region | 1.03 ± 0.24 | 0.90 ± 0.14 | -12.75% | 3.1 | 0.003 |
| Body Fat Content Distribution |  |  |  |  |  |
| Arm region | 0.11 ± 0.01 | 0.11 ± 0.01 | 3.12% | -1.5 | 0.147 |
| Leg region | 0.32 ± 0.04 | 0.37 ± 0.03 | 15.02% | -6.3 | <0.001 |
| Trunk region | 0.49 ± 0.07 | 0.46 ± 0.04 | -6.91% | 2.9 | 0.006 |
| Android region | 0.07 ± 0.02 | 0.06 ± 0.01 | -8.72% | 1.8 | 0.077 |
| Gynoid region | 0.16 ± 0.02 | 0.18 ± 0.01 | 10.67% | -5.9 | <0.001 |
| Visceral region | 0.03 ± 0.01 | 0.02 ± 0.01 | -44.67% | 6.0 | <0.001 |
| Corrected leg region | 0.16 ± 0.04 | 0.19 ± 0.03 | 19.31% | -4.5 | <0.001 |
| Lean Body Mass Content |  |  |  |  |  |
| Total body (kg) | 46.83 ± 5.66 | 45.19 ± 6.16 | -3.49% | 1.3 | 0.184 |
| Arm region (kg) | 5.34 ± 0.89 | 4.88 ± 0.71 | -8.65% | 2.9 | 0.005 |
| Leg region (kg) | 16.54 ± 2.40 | 16.27 ± 2.63 | -1.65% | 0.5 | 0.6 |
| Trunk region (kg) | 20.84 ± 2.44 | 20.01 ± 2.88 | -4.00% | 1.5 | 0.137 |
| Android region (kg) | 2.91 ± 0.35 | 2.82 ± 0.44 | -3.05% | 1.1 | 0.291 |
| Gynoid region (kg) | 7.12 ± 0.96 | 6.85 ± 1.09 | -3.75% | 1.3 | 0.213 |
| Corrected leg region (kg) | 9.43 ± 1.59 | 9.42 ± 1.69 | -0.07% | 0.02 | 0.985 |
| Android region/ Gynoid region | 0.41 ± 0.02 | 0.41 ± 0.03 | 0 | -0.5 | 0.609 |
| Lean Body Mass Content Percentage |  |  |  |  |  |
| Total body | 0.78 ± 0.07 | 0.74 ± 0.05 | -5.13% | 3.0 | 0.004 |
| Arm region | 0.79 ± 0.07 | 0.73 ± 0.06 | -7.59% | 4.2 | <0.001 |
| Leg region | 0.79 ± 0.06 | 0.73 ± 0.05 | -7.59% | 5.5 | <0.001 |
| Trunk region | 0.75 ± 0.09 | 0.73 ± 0.07 | -2.67% | 1.6 | 0.123 |
| Android region | 0.75 ± 0.11 | 0.73 ± 0.08 | -2.67% | 1.0 | 0.324 |
| Gynoid region | 0.77 ± 0.07 | 0.71 ± 0.06 | -7.79% | 4.8 | <0.001 |
| Corrected leg region | 0.45 ± 0.04 | 0.42 ± 0.03 | -6.43% | 3.6 | <0.001 |
| Android region/ Gynoid region | 0.98 ± 0.08 | 1.04 ± 0.06 | 6.12% | -4.3 | <0.001 |
| Lean Body Mass Content Distribution |  |  |  |  |  |
| Arm region | 0.1137 ± 0.01 | 0.1081 ± 0.01 | -4.93% | 3.3 | 0.001 |
| Leg region | 0.35 ± 0.01 | 0.36 ± 0.02 | 2.86% | -2.1 | 0.04 |
| Trunk region | 0.45 ± 0.01 | 0.44 ± 0.02 | -2.22% | 1.0 | 0.323 |
| Android region | 0.06 ± 0.00 | 0.06 ± 0.00 | 0 | -0.2 | 0.869 |
| Gynoid region | 0.15 ± 0.01 | 0.15 ± 0.01 | 0 | 0.4 | 0.717 |
| Corrected leg region | 0.20 ± 0.02 | 0.21 ± 0.02 | 3.61% | 2.0 | 0.044 |
| Body Mass Distribution |  |  |  |  |  |
| Arm region | 0.11 ± 0.01 | 0.11 ± 0.01 | -3.00% | 2.2 | 0.031 |
| Leg region | 0.34 ± 0.02 | 0.36 ± 0.02 | 5.01% | -5.2 | ＜0.001 |
| Trunk region | 0.46 ± 0.02 | 0.45 ± 0.02 | -2.52% | 3.0 | 0.003 |
| Android region | 0.06 ± 0.01 | 0.06 ± 0.01 | -2.79% | 1.5 | 0.131 |
| Gynoid region | 0.15 ± 0.01 | 0.16 ± 0.01 | 2.89% | -3.1 | 0.002 |
| Corrected leg region | 0.19 ± 0.02 | 0.20 ± 0.02 | 6..72% | 3.6 | <.001 |

**Table 2 Body fat and Lean body mass in tranwomen after stratification according to GAHT duration**

| The duration of treatment | **Stratification** | | | | | ***F*** | ***P*** | **Reference value cis women** | **Reference value cis men** |
| --- | --- | --- | --- | --- | --- | --- | --- | --- | --- |
|  | **0**  **(n = 40)** | **0-1**  **(n = 31)** | **1 -2**  **(n = 10)** | **2-3**  **(n = 10)** | **＞3**  **(n = 8)** |  |  |  |  |
| Body Fat Content |  |  |  |  |  |  |  |  |  |
| Total body (kg) | 13.95 ± 5.55 | 15.20 ± 5.87 | 18.89 ± 3.15 | 17.64 ± 5.99 | 18.52 ± 6.57 | 2.6 | 0.038 |  |  |
| Arm region (kg) | 1.48 ± 0.57 | 1.65 ± 0.63 | 2.12 ± 0.39 | 1.93 ± 0.61 | 2.09 ± 0.68 | 4.0 | 0.005 |  |  |
| Leg region (kg) | 4.32 ± 1.50 | 5.48 ± 2.15 | 7.09 ± 1.38 | 6.56 ± 2.14 | 6.75 ± 1.92 | 7.9 | <0.001 |  |  |
| Trunk region (kg) | 7.18 ± 3.61 | 7.11 ± 3.16 | 8.72 ± 1.71 | 8.17 ± 3.44 | 8.76 ± 3.97 | 0.9 | 0.463 |  |  |
| Android region (kg) | 1.05 ± 0.65 | 0.98 ± 0.54 | 1.22 ± 0.32 | 1.17 ± 0.69 | 1.29 ± 0.75 | 0.7 | 0.624 |  |  |
| Gynoid region (kg) | 2.20 ± 0.86 | 2.68 ± 1.07 | 3.30 ± 0.51 | 3.09 ± 1.09 | 3.13 ± 0.86 | 4.7 | 0.002 |  |  |
| Corrected leg region (kg) | 2.76 ± 1.12 | 2.12 ± 0.70 | 2.80 ± 1.14 | 3.79 ± 0.90 | 3.63 ± 1.08 | 10.6 | ＜0.001 |  |  |
| Android region/ Gynoid region | 0.44 ± 0.15 | 0.35 ± 0.06 | 0.37 ± 0.07 | 0.36 ± 0.10 | 0.39 ± 0.13 |  | 0.048 |  |  |
| Visceral region(kg) | 0.47 ± 0.37 | 0.28 ± 0.21 | 0.25 ± 0.12 | 0.32 ± 0.17 | 0.36 ± 0.28 |  | 0.057 |  |  |
| Body Fat Content Percentage |  |  |  |  |  |  |  |  |  |
| Total body | 0.22 ± 0.07 | 0.25 ± 0.05 | 0.29 ± 0.03 | 0.26 ± 0.06 | 0.29 ± 0.06 |  | 0.001 |  |  |
| Arm region | 0.21 ± 0.07 | 0.25 ± 0.06 | 0.29 ± 0.03 | 0.27 ± 0.06 | 0.31 ± 0.05 |  | <0.001 | 0.31^26^ | 0.14^26^ |
| Leg region | 0.21 ± 0.06 | 0.25 ± 0.05 | 0.30 ± 0.04 | 0.27 ± 0.05 | 0.29 ± 0.04 | 10.2 | <0.001 | 0.36^26^ | 0.18^26^ |
| Trunk region | 0.25 ± 0.09 | 0.26 ± 0.07 | 0.30 ± 0.03 | 0.27 ± 0.07 | 0.30 ± 0.08 |  | 0.03 | 0.36^27^ | 0.18^29^ |
| Android region | 0.25 ± 0.11 | 0.25 ± 0.08 | 0.30 ± 0.05 | 0.26 ± 0.09 | 0.31 ± 0.10 |  | 0.113 | 0.35^28^ | 0.25^13^ |
| Gynoid region | 0.23 ± 0.07 | 0.28 ± 0.06 | 0.32 ± 0.03 | 0.29 ± 0.06 | 0.32 ± 0.04 |  | <0.001 | 0.42^28^ | 0.24^13^ |
| Corrected leg region | 0.18 ± 0.05 | 0.23 ± 0.05 | 0.28 ± 0.05 | 0.26 ± 0.05 | 0.27 ± 0.05 | 12.4 | <0.001 |  |  |
| Android region/ Gynoid region | 1.03 ± 0.24 | 0.89 ± 0.12 | 0.92 ± 0.11 | 0.88 ± 0.16 | 0.94 ± 0.23 |  | 0.059 |  |  |
| Body Fat Content Distribution |  |  |  |  |  |  |  |  |  |
| Arm region | 0.11 ± 0.01 | 0.11 ± 0.01 | 0.11 ± 0.01 | 0.11 ± 0.01 | 0.11 ± 0.01 | 0.9 | 0.467 |  |  |
| Leg region | 0.32 ± 0.04 | 0.36 ± 0.03 | 0.37 ± 0.03 | 0.38 ± 0.04 | 0.37 ± 0.03 | 10.5 | <0.001 |  |  |
| Trunk region | 0.49 ± 0.07 | 0.46 ± 0.04 | 0.46 ± 0.04 | 0.45 ± 0.05 | 0.46 ± 0.05 |  | 0.145 |  |  |
| Android region | 0.07 ± 0.02 | 0.06 ± 0.01 | 0.06 ± 0.01 | 0.06 ± 0.01 | 0.07 ± 0.01 | 1.0 | 0.389 |  |  |
| Gynoid region | 0.16 ± 0.02 | 0.18 ± 0.01 | 0.18 ± 0.01 | 0.17 ± 0.02 | 0.17 ± 0.02 |  | <0.001 |  |  |
| Visceral region | 0.03 ± 0.01 | 0.02 ± 0.01 | 0.01 ± 0.01 | 0.02 ± 0.01 | 0.02 ± 0.01 | 11.2 | <0.001 |  |  |
| Corrected leg region | 0.16 ± 0.04 | 0.18 ± 0.03 | 0.20 ± 0.03 | 0.20 ± 0.04 | 0.20 ± 0.02 | 5.9 | ＜0.001 |  |  |
| Lean Body Mass Content |  |  |  |  |  |  |  |  |  |
| Total body (kg) | 46.83 ± 5.66 | 44.64 ± 6.93 | 45.78 ± 4.82 | 47.80 ± 4.63 | 43.32 ± 5.96 | 1.2 | 0.31 |  |  |
| Arm region (kg) | 5.34 ± 0.89 | 4.82 ± 0.78 | 5.05 ± 0.67 | 5.19 ± 0.44 | 4.52 ± 0.62 | 3.1 | 0.02 |  |  |
| Leg region (kg) | 16.54 ± 2.40 | 16.02 ± 3.07 | 16.55 ± 1.74 | 16.92 ± 1.98 | 16.07 ± 2.64 | 0.3 | 0.849 |  |  |
| Trunk region (kg) | 20.84 ± 2.44 | 19.73 ± 3.13 | 20.24 ± 2.54 | 21.53 ± 2.24 | 18.92 ± 2.64 | 1.8 | 0.134 |  |  |
| Android region (kg) | 2.91 ± 0.35 | 2.77 ± 0.49 | 2.81 ± 0.32 | 3.08 ± 0.39 | 2.68 ± 0.38 | 1.7 | 0.166 |  |  |
| Gynoid region (kg) | 7.12 ± 0.96 | 6.79 ± 1.23 | 6.85 ± 0.72 | 7.25 ± 0.92 | 6.58 ± 1.09 | 0.9 | 0.455 |  |  |
| Corrected leg region (kg) | 9.43 ± 1.59 | 9.23 ± 1.98 | 9.70 ± 1.17 | 9.66 ± 1.29 | 9.49 ± 1.65 | 0.2 | 0.923 |  |  |
| Android region/ Gynoid region | 0.41 ± 0.02 | 0.41 ± 0.03 | 0.41 ± 0.02 | 0.43 ± 0.02 | 0.41 ± 0.03 | 0.9 | 0.472 |  |  |
| Lean Body Mass Content Percentage |  |  |  |  |  |  |  |  |  |
| Total body | 0.78 ± 0.07 | 0.75 ± 0.05 | 0.71 ± 0.03 | 0.74 ± 0.06 | 0.71 ± 0.06 |  | 0.001 |  |  |
| Arm region | 0.79 ± 0.07 | 0.75 ± 0.06 | 0.71 ± 0.03 | 0.73 ± 0.06 | 0.69 ± 0.05 |  | <0.001 | 0.61^26^ | 0.81^26^ |
| Leg region | 0.79 ± 0.06 | 0.75 ± 0.05 | 0.70 ± 0.04 | 0.73 ± 0.05 | 0.71 ± 0.04 | 10.2 | <0.001 | 0.62^26^ | 0.78^26^ |
| Trunk region | 0.75 ± 0.09 | 0.74 ± 0.07 | 0.70 ± 0.03 | 0.73 ± 0.07 | 0.70 ± 0.08 |  | 0.03 | 0.64^27^ | 0.76^29^ |
| Android region | 0.75 ± 0.11 | 0.75 ± 0.08 | 0.70 ± 0.05 | 0.74 ± 0.09 | 0.69 ± 0.10 |  | 0.113 |  |  |
| Gynoid region | 0.77 ± 0.07 | 0.72 ± 0.06 | 0.68 ± 0.03 | 0.71 ± 0.06 | 0.68 ± 0.04 |  | <0.001 |  |  |
| Corrected leg region | 0.45 ± 0.04 | 0.43 ± 0.03 | 0.41 ± 0.02 | 0.41 ± 0.04 | 0.42 ± 0.03 | 4.4 | 0.003 |  |  |
| Android region/ Gynoid region | 0.98 ± 0.08 | 1.04 ± 0.04 | 1.04 ± 0.05 | 1.04 ± 0.07 | 1.01 ± 0.10 |  | 0.009 |  |  |
| Lean Body Mass Content Distribution |  |  |  |  |  |  |  |  |  |
| Arm region | 0.11 ± 0.01 | 0.11 ± 0.01 | 0.11 ± 0.01 | 0.11 ± 0.01 | 0.10 ± 0.01 | 3.2 | 0.017 |  |  |
| Leg region | 0.35 ± 0.01 | 0.36 ± 0.02 | 0.36 ± 0.01 | 0.35 ± 0.01 | 0.37 ± 0.02 | 2.5 | 0.046 |  |  |
| Trunk region | 0.45 ± 0.01 | 0.44 ± 0.02 | 0.44 ± 0.02 | 0.45 ± 0.01 | 0.44 ± 0.01 | 1.3 | 0.259 |  |  |
| Android region | 0.06 ± 0.004 | 0.06 ± 0.005 | 0.06 ± 0.002 | 0.06 ± 0.003 | 0.06 ± 0.005 |  | 0.101 |  |  |
| Gynoid region | 0.15 ± 0.01 | 0.15 ± 0.01 | 0.15 ± 0.01 | 0.15 ± 0.01 | 0.15 ± 0.01 | 0.2 | 0.956 |  |  |
| Corrected leg region | 0.20 ± 0.02 | 0.21 ± 0.02 | 0.21 ± 0.01 | 0.20 ± 0.02 | 0.22 ± 0.02 | 2.3 | 0.062 |  |  |
| Body Mass Distribution |  |  |  |  |  |  |  |  |  |
| Arm region | 0.11 ± 0.01 | 0.11 ± 0.01 | 0.11 ± 0.01 | 0.11 ± 0.01 | 0.11 ± 0.01 | 1.5 | 0.210 |  |  |
| Leg region | 0.34 ± 0.02 | 0.36 ± 0.02 | 0.37 ± 0.01 | 0.36 ± 0.01 | 0.37 ± 0.01 | 7.8 | ＜0.001 |  |  |
| Trunk region | 0.46 ± 0.02 | 0.45 ± 0.02 | 0.45 ± 0.02 | 0.45 ± 0.02 | 0.45 ± 0.02 | 2.4 | 0.056 |  |  |
| Android region | 0.06 ± 0.01 | 0.06 ± 0.01 | 0.06 ± 0.003 | 0.06 ± 0.01 | 0.06 ± 0.01 | 0.9 | 0.471 |  |  |
| Gynoid region | 0.15 ± 0.01 | 0.16 ± 0.01 | 0.16 ± 0.01 | 0.16 ± 0.01 | 0.16 ± 0.01 | 2.4 | 0.054 |  |  |
| Corrected leg region | 0.19 ± 0.02 | 0.20 ± 0.02 | 0.21 ± 0.01 | 0.20 ± 0.02 | 0.21 ± 0.01 | 4.2 | 0.003 |  |  |

0: Not received GAHT; 0-1: 0＜Duration of treatment≤1 year; 1-2: 1 year＜Duration of treatment≤2 years; 2-3: 2 years＜Duration of treatment≤3 years; ＞3: Duration of treatment＞3 years

Age categories for reference values; Women: ^26^ category 20-29 years old, ^27^ median (range) 31 (18-62) years old, ^28^ mean (SD) 20.8 (1.9) years old; Men: ^26^ category 20-29 years old, ^29^ median (range) 31 (18-55) years old, ^13^ mean (SD) 21.3 (2.2).

**Table 3 Results of multiple comparisons of body fat and lean body mass in transwomen after stratification according to GAHT duration**

|  | **GAHT duration** | **GAHT duration** | ***P*** | ***95%Cl*** |
| --- | --- | --- | --- | --- |
| Body Fat Content |  |  |  |  |
| Arm region (kg) | 0 | 1-2 | 0.026 | (-1.24, -0.05) |
| Leg region (kg) | 0 | 1-2 | ＜0.001 | (-4.62, -0.93) |
|  | 0 | 2-3 | 0.007 | (-4.09, -0.40) |
|  | 0 | ＞3 | 0.008 | (-4.46, -0.41) |
| Gynoid region (kg) | 0 | 1-2 | 0.012 | (-2.05, -0.16) |
| Corrected leg region (kg) | 0 | 0-1 | 0.035 | (-1.33, -0.03) |
|  | 0 | 1-2 | ＜0.001 | (-2.64,-0.71) |
|  | 0 | 2-3 | 0.001 | (-2.31, -0.38) |
|  | 0 | ＞3 | 0.001 | (-2.56, -0.45) |
| Android region/ Gynoid region | 0 | 0-1 | 0.009 | (0.02, 0.16) |
| Body Fat Mass Percentage |  |  |  |  |
| Total body | 0 | 1-2 | ＜0.001 | (-0.11, -0.03) |
|  | 0-1 | 1-2 | 0.022 | (-0.08, -0.005) |
| Arm region | 0 | 1-2 | ＜0.001 | (-0.12, -0.04) |
|  | 0 | ＞3 | 0.005 | (-0.16, -0.03) |
|  | 0-1 | 1-2 | 0.048 | (-0.09, -0.0013) |
| Leg region | 0 | 0-1 | 0.011 | (-0.08, -0.01) |
|  | 0 | 1-2 | ＜0.001 | (-0.15, -0.04) |
|  | 0 | 2-3 | 0.004 | (-0.12, -0.01) |
|  | 0 | ＞3 | 0.001 | (-0.15, -0.03) |
| Trunk region | 0 | 1-2 | 0.032 | (-0.11, -0.003) |
| Gynoid region | 0 | 0-1 | 0.042 | (-0.09, -0.001) |
|  | 0 | 1-2 | ＜0.001 | (-0.14, -0.05) |
|  | 0 | ＞3 | 0.001 | (-0.14, -0.03) |
|  | 0-1 | 1-2 | 0.030 | (-0.09, -0.004) |
| Corrected leg region | 0 | 0-1 | 0.006 | (-0.08, -0.01) |
|  | 0 | 1-2 | ＜0.001 | (-0.15, -0.04) |
|  | 0 | 2-3 | 0.001 | (-0.13, -0.02) |
|  | 0 | ＞3 | ＜0.001 | (-0.15, -0.03) |
| Body Fat Mass Distribution |  |  |  |  |
| Leg region | 0 | 0-1 | ＜0.001 | (-0.07, -0.02) |
|  | 0 | 1-2 | ＜0.001 | (-0.09, -0.02) |
|  | 0 | 2-3 | ＜0.001 | (-0.09, -0.02) |
|  | 0 | ＞3 | 0.003 | (-0.10, -0.01) |
| Gynoid region | 0 | 0-1 | ＜0.001 | (-0.03, -0.01) |
|  | 0 | 1-2 | 0.003 | (-0.03, -0.005) |
| Corrected leg region | 0 | 0-1 | 0.039 | (-0.05, -0.0007) |
|  | 0 | 1-2 | 0.016 | (-0.07, -0.005) |
|  | 0 | 2-3 | 0.011 | (-0.07, -0.01) |
|  | 0 | ＞3 | 0.038 | (-0.08, -0.001) |
| Lean Body Mass Percentage |  |  |  |  |
| Total body | 0 | 1-2 | ＜0.001 | (0.03, 0.11) |
|  | 0-1 | 1-2 | 0.022 | (0.005, 0.08) |
| Arm region | 0 | 1-2 | ＜0.001 | (0.04, 0.12) |
|  | 0 | ＞3 | 0.005 | (0.03, 0.16) |
|  | 0-1 | 1-2 | 0.048 | (0.0013, 0.09) |
| Leg region | 0 | 0-1 | 0.011 | (0.01, 0.08) |
|  | 0 | 1-2 | ＜0.001 | (0.04, 0.15) |
|  | 0 | 2-3 | 0.004 | (0.01, 0.12) |
|  | 0 | ＞3 | 0.001 | (0.03, 0.15) |
| Trunk region | 0 | 1-2 | 0.032 | (0.003, 0.11) |
| Gynoid region | 0 | 0-1 | 0.042 | (0.001, 0.09) |
|  | 0 | 1-2 | ＜0.001 | (0.05, 0.14) |
|  | 0 | ＞3 | 0.001 | (0.03, 0.14) |
|  | 0-1 | 1-2 | 0.030 | (0.004, 0.09) |
| Corrected leg region | 0 | 1-2 | 0.024 | (0.003, 0.08) |
| Android region/ Gynoid region | 0 | 0-1 | 0.001 | (-0.10, 0.02) |
| Total Body Mass Distribution |  |  |  |  |
| Leg region | 0 | 0-1 | 0.004 | (-0.03, -0.003) |
|  | 0 | 1-2 | 0.002 | (-0.04, -0.01) |
|  | 0 | ＞3 | 0.001 | (-0.04, -0.01) |
| Corrected leg region | 0 | 1-2 | 0.045 | (-0.04, -0.0002) |
|  | 0 | ＞3 | 0.019 | (-0.04, -0.002) |
| Blood glucose(mmol/L) | 0 | 1-2 | 0.035 | (-0.95, -0.03) |
|  | 0 | 2-3 | 0.050 | (--2.24, -0.0004) |

0: Not received GAHT; 0-1: 0＜Duration of treatment≤1 year; 1-2: 1 year＜Duration of treatment≤2 years; 2-3: 2 years＜Duration of treatment≤3 years; ＞3: Duration of treatment＞3 years
