# Supplementary material for: Effects of gender-affirming hormone therapy on body fat: a retrospective case‒control study in Chinese transwomen
Source: Lipids Health Dis. 2024 May 17;23:146. doi: 10.1186/s12944-024-02131-y (PMC11100057; doi:10.1186/s12944-024-02131-y)
Supplement: Supplementary file 3 — Supplementary Material 3 [file 12944_2024_2131_MOESM3_ESM.docx]

**Supplementary materia**

Table1-3:Values are given as mean ± SD; GAHT：Gender-affirming hormone therapy

**Table 1** Body fat and Lean body mass in transwomen

|  | Categorization | | magnitude of change | *t* | *P* |
| --- | --- | --- | --- | --- | --- |
|  | Not received GAHT (n = 40) | Received GAHT  (n = 59) |  |  |  |
| Body Fat Content |  |  |  |  |  |
| Total body (kg) | 13.95 ± 5.55 | 16.69 ± 5.72 | 19.65% | -2.4 | 0.02 |
| Arm region (kg) | 1.48 ± 0.57 | 1.83 ± 0.62 | 24.02% | -2.9 | 0.005 |
| Leg region (kg) | 4.32 ± 1.50 | 6.11 ± 2.08 | 41.47% | -4.7 | <0.001 |
| Trunk region (kg) | 7.18 ± 3.61 | 7.79 ± 3.14 | 8.39% | -0.9 | 0.38 |
| Android region (kg) | 1.05 ± 0.65 | 1.10 ± 0.57 | 4.89% | -0.4 | 0.68 |
| Gynoid region (kg) | 2.20 ± 0.86 | 2.92 ± 0.98 | 32.60% | -3.7 | <0.001 |
| Corrected leg region(kg) | 2.12 ± 0.70 | 3.19 ± 1.15 | 50.69% | -5.8 | <0.001 |
| Android region/ Gynoid region | 0.44 ± 0.15 | 0.36 ± 0.08 | -18.30% | 3.1 | 0.003 |
| Visceral region(kg) | 0.47 ± 0.37 | 0.29 ± 0.20 | -37.49% | 2.8 | 0.008 |
| Body Fat Content Percentage |  |  |  |  |  |
| Total body | 0.22 ± 0.07 | 0.26 ± 0.05 | 17.63% | -3.0 | 0.004 |
| Arm region | 0.21 ± 0.07 | 0.27 ± 0.06 | 25.19% | -4.2 | <0.001 |
| Leg region | 0.21 ± 0.06 | 0.27 ± 0.05 | 30.39% | -5.5 | <0.001 |
| Trunk region | 0.25 ± 0.09 | 0.27 ± 0.07 | 10.84% | -1.6 | 0.123 |
| Android region | 0.25 ± 0.11 | 0.27 ± 0.08 | 8.25% | -1.0 | 0.324 |
| Gynoid region | 0.23 ± 0.07 | 0.29 ± 0.06 | 26.79% | -4.8 | <0.001 |
| Corrected leg region | 0.18 ± 0.05 | 0.25 ± 0.05 | 34.90% | -5.9 | <0.001 |
| Android region/ Gynoid region | 1.03 ± 0.24 | 0.90 ± 0.14 | -12.75% | 3.1 | 0.003 |
| Body Fat Content Distribution |  |  |  |  |  |
| Arm region | 0.11 ± 0.01 | 0.11 ± 0.01 | 3.12% | -1.5 | 0.147 |
| Leg region | 0.32 ± 0.04 | 0.37 ± 0.03 | 15.02% | -6.3 | <0.001 |
| Trunk region | 0.49 ± 0.07 | 0.46 ± 0.04 | -6.91% | 2.9 | 0.006 |
| Android region | 0.07 ± 0.02 | 0.06 ± 0.01 | -8.72% | 1.8 | 0.077 |
| Gynoid region | 0.16 ± 0.02 | 0.18 ± 0.01 | 10.67% | -5.9 | <0.001 |
| Visceral region | 0.03 ± 0.01 | 0.02 ± 0.01 | -44.67% | 6.0 | <0.001 |
| Corrected leg region | 0.16 ± 0.04 | 0.19 ± 0.03 | 19.31% | -4.5 | <0.001 |
| Lean Body Mass Content |  |  |  |  |  |
| Total body (kg) | 46.83 ± 5.66 | 45.19 ± 6.16 | -3.49% | 1.3 | 0.184 |
| Arm region (kg) | 5.34 ± 0.89 | 4.88 ± 0.71 | -8.65% | 2.9 | 0.005 |
| Leg region (kg) | 16.54 ± 2.40 | 16.27 ± 2.63 | -1.65% | 0.5 | 0.6 |
| Trunk region (kg) | 20.84 ± 2.44 | 20.01 ± 2.88 | -4.00% | 1.5 | 0.137 |
| Android region (kg) | 2.91 ± 0.35 | 2.82 ± 0.44 | -3.05% | 1.1 | 0.291 |
| Gynoid region (kg) | 7.12 ± 0.96 | 6.85 ± 1.09 | -3.75% | 1.3 | 0.213 |
| Corrected leg region (kg) | 9.43 ± 1.59 | 9.42 ± 1.69 | -0.07% | 0.02 | 0.985 |
| Android region/ Gynoid region | 0.41 ± 0.02 | 0.41 ± 0.03 | 0 | -0.5 | 0.609 |
| Lean Body Mass Content Percentage |  |  |  |  |  |
| Total body | 0.78 ± 0.07 | 0.74 ± 0.05 | -5.13% | 3.0 | 0.004 |
| Arm region | 0.79 ± 0.07 | 0.73 ± 0.06 | -7.59% | 4.2 | <0.001 |
| Leg region | 0.79 ± 0.06 | 0.73 ± 0.05 | -7.59% | 5.5 | <0.001 |
| Trunk region | 0.75 ± 0.09 | 0.73 ± 0.07 | -2.67% | 1.6 | 0.123 |
| Android region | 0.75 ± 0.11 | 0.73 ± 0.08 | -2.67% | 1.0 | 0.324 |
| Gynoid region | 0.77 ± 0.07 | 0.71 ± 0.06 | -7.79% | 4.8 | <0.001 |
| Corrected leg region | 0.45 ± 0.04 | 0.42 ± 0.03 | -6.43% | 3.6 | <0.001 |
| Android region/ Gynoid region | 0.98 ± 0.08 | 1.04 ± 0.06 | 6.12% | -4.3 | <0.001 |
| Lean Body Mass Content Distribution |  |  |  |  |  |
| Arm region | 0.1137 ± 0.01 | 0.1081 ± 0.01 | -4.93% | 3.3 | 0.001 |
| Leg region | 0.35 ± 0.01 | 0.36 ± 0.02 | 2.86% | -2.1 | 0.04 |
| Trunk region | 0.45 ± 0.01 | 0.44 ± 0.02 | -2.22% | 1.0 | 0.323 |
| Android region | 0.06 ± 0.00 | 0.06 ± 0.00 | 0 | -0.2 | 0.869 |
| Gynoid region | 0.15 ± 0.01 | 0.15 ± 0.01 | 0 | 0.4 | 0.717 |
| Corrected leg region | 0.20 ± 0.02 | 0.21 ± 0.02 | 3.61% | 2.0 | 0.044 |
| Body Mass Distribution |  |  |  |  |  |
| Arm region | 0.11 ± 0.01 | 0.11 ± 0.01 | -3.00% | 2.2 | 0.031 |
| Leg region | 0.34 ± 0.02 | 0.36 ± 0.02 | 5.01% | -5.2 | ＜0.001 |
| Trunk region | 0.46 ± 0.02 | 0.45 ± 0.02 | -2.52% | 3.0 | 0.003 |
| Android region | 0.06 ± 0.01 | 0.06 ± 0.01 | -2.79% | 1.5 | 0.131 |
| Gynoid region | 0.15 ± 0.01 | 0.16 ± 0.01 | 2.89% | -3.1 | 0.002 |
| Corrected leg region | 0.19 ± 0.02 | 0.20 ± 0.02 | 6..72% | 3.6 | <.001 |

**Table 2** Body fat and Lean body mass in tranwomen after stratification according to GAHT duration

| The duration of treatment | Stratification | | | | | *F* | *P* | Reference value cis women | Reference value cis men |
| --- | --- | --- | --- | --- | --- | --- | --- | --- | --- |
|  | 0  (n = 40) | 0-1  (n = 31) | 1 -2  (n = 10) | 2-3  (n = 10) | ＞3  (n = 8) |  |  |  |  |
| Body Fat Content |  |  |  |  |  |  |  |  |  |
| Total body (kg) | 13.95 ± 5.55 | 15.20 ± 5.87 | 18.89 ± 3.15 | 17.64 ± 5.99 | 18.52 ± 6.57 | 2.6 | 0.038 |  |  |
| Arm region (kg) | 1.48 ± 0.57 | 1.65 ± 0.63 | 2.12 ± 0.39 | 1.93 ± 0.61 | 2.09 ± 0.68 | 4.0 | 0.005 |  |  |
| Leg region (kg) | 4.32 ± 1.50 | 5.48 ± 2.15 | 7.09 ± 1.38 | 6.56 ± 2.14 | 6.75 ± 1.92 | 7.9 | <0.001 |  |  |
| Trunk region (kg) | 7.18 ± 3.61 | 7.11 ± 3.16 | 8.72 ± 1.71 | 8.17 ± 3.44 | 8.76 ± 3.97 | 0.9 | 0.463 |  |  |
| Android region (kg) | 1.05 ± 0.65 | 0.98 ± 0.54 | 1.22 ± 0.32 | 1.17 ± 0.69 | 1.29 ± 0.75 | 0.7 | 0.624 |  |  |
| Gynoid region (kg) | 2.20 ± 0.86 | 2.68 ± 1.07 | 3.30 ± 0.51 | 3.09 ± 1.09 | 3.13 ± 0.86 | 4.7 | 0.002 |  |  |
| Corrected leg region (kg) | 2.76 ± 1.12 | 2.12 ± 0.70 | 2.80 ± 1.14 | 3.79 ± 0.90 | 3.63 ± 1.08 | 10.6 | ＜0.001 |  |  |
| Android region/ Gynoid region | 0.44 ± 0.15 | 0.35 ± 0.06 | 0.37 ± 0.07 | 0.36 ± 0.10 | 0.39 ± 0.13 | **‒** | 0.048 |  |  |
| Visceral region(kg) | 0.47 ± 0.37 | 0.28 ± 0.21 | 0.25 ± 0.12 | 0.32 ± 0.17 | 0.36 ± 0.28 | **‒** | 0.057 |  |  |
| Body Fat Content Percentage |  |  |  |  |  |  |  |  |  |
| Total body | 0.22 ± 0.07 | 0.25 ± 0.05 | 0.29 ± 0.03 | 0.26 ± 0.06 | 0.29 ± 0.06 | **‒** | 0.001 |  |  |
| Arm region | 0.21 ± 0.07 | 0.25 ± 0.06 | 0.29 ± 0.03 | 0.27 ± 0.06 | 0.31 ± 0.05 | **‒** | <0.001 | 0.31^30^ | 0.14^30^ |
| Leg region | 0.21 ± 0.06 | 0.25 ± 0.05 | 0.30 ± 0.04 | 0.27 ± 0.05 | 0.29 ± 0.04 | 10.2 | <0.001 | 0.36^30^ | 0.18^30^ |
| Trunk region | 0.25 ± 0.09 | 0.26 ± 0.07 | 0.30 ± 0.03 | 0.27 ± 0.07 | 0.30 ± 0.08 | **‒** | 0.03 | 0.36^31^ | 0.18^33^ |
| Android region | 0.25 ± 0.11 | 0.25 ± 0.08 | 0.30 ± 0.05 | 0.26 ± 0.09 | 0.31 ± 0.10 | **‒** | 0.113 | 0.35^15^ | 0.25^32^ |
| Gynoid region | 0.23 ± 0.07 | 0.28 ± 0.06 | 0.32 ± 0.03 | 0.29 ± 0.06 | 0.32 ± 0.04 | **‒** | <0.001 | 0.42^15^ | 0.24^32^ |
| Corrected leg region | 0.18 ± 0.05 | 0.23 ± 0.05 | 0.28 ± 0.05 | 0.26 ± 0.05 | 0.27 ± 0.05 | 12.4 | <0.001 |  |  |
| Android region/ Gynoid region | 1.03 ± 0.24 | 0.89 ± 0.12 | 0.92 ± 0.11 | 0.88 ± 0.16 | 0.94 ± 0.23 | **‒** | 0.059 |  |  |
| Body Fat Content Distribution |  |  |  |  |  |  |  |  |  |
| Arm region | 0.11 ± 0.01 | 0.11 ± 0.01 | 0.11 ± 0.01 | 0.11 ± 0.01 | 0.11 ± 0.01 | 0.9 | 0.467 |  |  |
| Leg region | 0.32 ± 0.04 | 0.36 ± 0.03 | 0.37 ± 0.03 | 0.38 ± 0.04 | 0.37 ± 0.03 | 10.5 | <0.001 |  |  |
| Trunk region | 0.49 ± 0.07 | 0.46 ± 0.04 | 0.46 ± 0.04 | 0.45 ± 0.05 | 0.46 ± 0.05 | **‒** | 0.145 |  |  |
| Android region | 0.07 ± 0.02 | 0.06 ± 0.01 | 0.06 ± 0.01 | 0.06 ± 0.01 | 0.07 ± 0.01 | 1.0 | 0.389 |  |  |
| Gynoid region | 0.16 ± 0.02 | 0.18 ± 0.01 | 0.18 ± 0.01 | 0.17 ± 0.02 | 0.17 ± 0.02 | **‒** | <0.001 |  |  |
| Visceral region | 0.03 ± 0.01 | 0.02 ± 0.01 | 0.01 ± 0.01 | 0.02 ± 0.01 | 0.02 ± 0.01 | 11.2 | <0.001 |  |  |
| Corrected leg region | 0.16 ± 0.04 | 0.18 ± 0.03 | 0.20 ± 0.03 | 0.20 ± 0.04 | 0.20 ± 0.02 | 5.9 | ＜0.001 |  |  |
| Lean Body Mass Content |  |  |  |  |  |  |  |  |  |
| Total body (kg) | 46.83 ± 5.66 | 44.64 ± 6.93 | 45.78 ± 4.82 | 47.80 ± 4.63 | 43.32 ± 5.96 | 1.2 | 0.31 |  |  |
| Arm region (kg) | 5.34 ± 0.89 | 4.82 ± 0.78 | 5.05 ± 0.67 | 5.19 ± 0.44 | 4.52 ± 0.62 | 3.1 | 0.02 |  |  |
| Leg region (kg) | 16.54 ± 2.40 | 16.02 ± 3.07 | 16.55 ± 1.74 | 16.92 ± 1.98 | 16.07 ± 2.64 | 0.3 | 0.849 |  |  |
| Trunk region (kg) | 20.84 ± 2.44 | 19.73 ± 3.13 | 20.24 ± 2.54 | 21.53 ± 2.24 | 18.92 ± 2.64 | 1.8 | 0.134 |  |  |
| Android region (kg) | 2.91 ± 0.35 | 2.77 ± 0.49 | 2.81 ± 0.32 | 3.08 ± 0.39 | 2.68 ± 0.38 | 1.7 | 0.166 |  |  |
| Gynoid region (kg) | 7.12 ± 0.96 | 6.79 ± 1.23 | 6.85 ± 0.72 | 7.25 ± 0.92 | 6.58 ± 1.09 | 0.9 | 0.455 |  |  |
| Corrected leg region (kg) | 9.43 ± 1.59 | 9.23 ± 1.98 | 9.70 ± 1.17 | 9.66 ± 1.29 | 9.49 ± 1.65 | 0.2 | 0.923 |  |  |
| Android region/ Gynoid region | 0.41 ± 0.02 | 0.41 ± 0.03 | 0.41 ± 0.02 | 0.43 ± 0.02 | 0.41 ± 0.03 | 0.9 | 0.472 |  |  |
| Lean Body Mass Content Percentage |  |  |  |  |  |  |  |  |  |
| Total body | 0.78 ± 0.07 | 0.75 ± 0.05 | 0.71 ± 0.03 | 0.74 ± 0.06 | 0.71 ± 0.06 | **‒** | 0.001 |  |  |
| Arm region | 0.79 ± 0.07 | 0.75 ± 0.06 | 0.71 ± 0.03 | 0.73 ± 0.06 | 0.69 ± 0.05 | **‒** | <0.001 | 0.61^30^ | 0.81^30^ |
| Leg region | 0.79 ± 0.06 | 0.75 ± 0.05 | 0.70 ± 0.04 | 0.73 ± 0.05 | 0.71 ± 0.04 | 10.2 | <0.001 | 0.62^30^ | 0.78^30^ |
| Trunk region | 0.75 ± 0.09 | 0.74 ± 0.07 | 0.70 ± 0.03 | 0.73 ± 0.07 | 0.70 ± 0.08 | **‒** | 0.03 | 0.64^31^ | 0.76^33^ |
| Android region | 0.75 ± 0.11 | 0.75 ± 0.08 | 0.70 ± 0.05 | 0.74 ± 0.09 | 0.69 ± 0.10 | **‒** | 0.113 |  |  |
| Gynoid region | 0.77 ± 0.07 | 0.72 ± 0.06 | 0.68 ± 0.03 | 0.71 ± 0.06 | 0.68 ± 0.04 | **‒** | <0.001 |  |  |
| Corrected leg region | 0.45 ± 0.04 | 0.43 ± 0.03 | 0.41 ± 0.02 | 0.41 ± 0.04 | 0.42 ± 0.03 | 4.4 | 0.003 |  |  |
| Android region/ Gynoid region | 0.98 ± 0.08 | 1.04 ± 0.04 | 1.04 ± 0.05 | 1.04 ± 0.07 | 1.01 ± 0.10 | **‒** | 0.009 |  |  |
| Lean Body Mass Content Distribution |  |  |  |  |  |  |  |  |  |
| Arm region | 0.11 ± 0.01 | 0.11 ± 0.01 | 0.11 ± 0.01 | 0.11 ± 0.01 | 0.10 ± 0.01 | 3.2 | 0.017 |  |  |
| Leg region | 0.35 ± 0.01 | 0.36 ± 0.02 | 0.36 ± 0.01 | 0.35 ± 0.01 | 0.37 ± 0.02 | 2.5 | 0.046 |  |  |
| Trunk region | 0.45 ± 0.01 | 0.44 ± 0.02 | 0.44 ± 0.02 | 0.45 ± 0.01 | 0.44 ± 0.01 | 1.3 | 0.259 |  |  |
| Android region | 0.06 ± 0.004 | 0.06 ± 0.005 | 0.06 ± 0.002 | 0.06 ± 0.003 | 0.06 ± 0.005 | **‒** | 0.101 |  |  |
| Gynoid region | 0.15 ± 0.01 | 0.15 ± 0.01 | 0.15 ± 0.01 | 0.15 ± 0.01 | 0.15 ± 0.01 | 0.2 | 0.956 |  |  |
| Corrected leg region | 0.20 ± 0.02 | 0.21 ± 0.02 | 0.21 ± 0.01 | 0.20 ± 0.02 | 0.22 ± 0.02 | 2.3 | 0.062 |  |  |
| Body Mass Distribution |  |  |  |  |  |  |  |  |  |
| Arm region | 0.11 ± 0.01 | 0.11 ± 0.01 | 0.11 ± 0.01 | 0.11 ± 0.01 | 0.11 ± 0.01 | 1.5 | 0.210 |  |  |
| Leg region | 0.34 ± 0.02 | 0.36 ± 0.02 | 0.37 ± 0.01 | 0.36 ± 0.01 | 0.37 ± 0.01 | 7.8 | ＜0.001 |  |  |
| Trunk region | 0.46 ± 0.02 | 0.45 ± 0.02 | 0.45 ± 0.02 | 0.45 ± 0.02 | 0.45 ± 0.02 | 2.4 | 0.056 |  |  |
| Android region | 0.06 ± 0.01 | 0.06 ± 0.01 | 0.06 ± 0.003 | 0.06 ± 0.01 | 0.06 ± 0.01 | 0.9 | 0.471 |  |  |
| Gynoid region | 0.15 ± 0.01 | 0.16 ± 0.01 | 0.16 ± 0.01 | 0.16 ± 0.01 | 0.16 ± 0.01 | 2.4 | 0.054 |  |  |
| Corrected leg region | 0.19 ± 0.02 | 0.20 ± 0.02 | 0.21 ± 0.01 | 0.20 ± 0.02 | 0.21 ± 0.01 | 4.2 | 0.003 |  |  |

0: Not received GAHT; 0-1: 0<Duration of treatment≤1 year; 1-2: 1 year<Duration of treatment≤2 years; 2-3: 2 years<Duration of treatment≤3 years; >3: Duration of treatment>3 years

Age categories for reference values; Women: ^30^ category 20-29 years old, ^31^ median (range) 31 (18-62) years old, ^15^ mean (SD) 20.8 (1.9) years old; Men: ^30^ category 20-29 years old, ^33^ median (range) 31 (18-55) years old, ^32^ mean (SD) 21.3 (2.2).

**Table 3** Results of multiple comparisons of body fat and lean body mass in transwomen after stratification according to GAHT duration

|  | GAHT duration | GAHT duration | *P* | *95%Cl* |
| --- | --- | --- | --- | --- |
| Body Fat Content |  |  |  |  |
| Arm region (kg) | 0 | 1-2 | 0.026 | (-1.24, -0.05) |
| Leg region (kg) | 0 | 1-2 | ＜0.001 | (-4.62, -0.93) |
|  | 0 | 2-3 | 0.007 | (-4.09, -0.40) |
|  | 0 | ＞3 | 0.008 | (-4.46, -0.41) |
| Gynoid region (kg) | 0 | 1-2 | 0.012 | (-2.05, -0.16) |
| Corrected leg region (kg) | 0 | 0-1 | 0.035 | (-1.33, -0.03) |
|  | 0 | 1-2 | ＜0.001 | (-2.64,-0.71) |
|  | 0 | 2-3 | 0.001 | (-2.31, -0.38) |
|  | 0 | ＞3 | 0.001 | (-2.56, -0.45) |
| Android region/ Gynoid region | 0 | 0-1 | 0.009 | (0.02, 0.16) |
| Body Fat Mass Percentage |  |  |  |  |
| Total body | 0 | 1-2 | ＜0.001 | (-0.11, -0.03) |
|  | 0-1 | 1-2 | 0.022 | (-0.08, -0.005) |
| Arm region | 0 | 1-2 | ＜0.001 | (-0.12, -0.04) |
|  | 0 | ＞3 | 0.005 | (-0.16, -0.03) |
|  | 0-1 | 1-2 | 0.048 | (-0.09, -0.0013) |
| Leg region | 0 | 0-1 | 0.011 | (-0.08, -0.01) |
|  | 0 | 1-2 | ＜0.001 | (-0.15, -0.04) |
|  | 0 | 2-3 | 0.004 | (-0.12, -0.01) |
|  | 0 | ＞3 | 0.001 | (-0.15, -0.03) |
| Trunk region | 0 | 1-2 | 0.032 | (-0.11, -0.003) |
| Gynoid region | 0 | 0-1 | 0.042 | (-0.09, -0.001) |
|  | 0 | 1-2 | ＜0.001 | (-0.14, -0.05) |
|  | 0 | ＞3 | 0.001 | (-0.14, -0.03) |
|  | 0-1 | 1-2 | 0.030 | (-0.09, -0.004) |
| Corrected leg region | 0 | 0-1 | 0.006 | (-0.08, -0.01) |
|  | 0 | 1-2 | ＜0.001 | (-0.15, -0.04) |
|  | 0 | 2-3 | 0.001 | (-0.13, -0.02) |
|  | 0 | ＞3 | ＜0.001 | (-0.15, -0.03) |
| Body Fat Mass Distribution |  |  |  |  |
| Leg region | 0 | 0-1 | ＜0.001 | (-0.07, -0.02) |
|  | 0 | 1-2 | ＜0.001 | (-0.09, -0.02) |
|  | 0 | 2-3 | ＜0.001 | (-0.09, -0.02) |
|  | 0 | ＞3 | 0.003 | (-0.10, -0.01) |
| Gynoid region | 0 | 0-1 | ＜0.001 | (-0.03, -0.01) |
|  | 0 | 1-2 | 0.003 | (-0.03, -0.005) |
| Corrected leg region | 0 | 0-1 | 0.039 | (-0.05, -0.0007) |
|  | 0 | 1-2 | 0.016 | (-0.07, -0.005) |
|  | 0 | 2-3 | 0.011 | (-0.07, -0.01) |
|  | 0 | ＞3 | 0.038 | (-0.08, -0.001) |
| Lean Body Mass Percentage |  |  |  |  |
| Total body | 0 | 1-2 | ＜0.001 | (0.03, 0.11) |
|  | 0-1 | 1-2 | 0.022 | (0.005, 0.08) |
| Arm region | 0 | 1-2 | ＜0.001 | (0.04, 0.12) |
|  | 0 | ＞3 | 0.005 | (0.03, 0.16) |
|  | 0-1 | 1-2 | 0.048 | (0.0013, 0.09) |
| Leg region | 0 | 0-1 | 0.011 | (0.01, 0.08) |
|  | 0 | 1-2 | ＜0.001 | (0.04, 0.15) |
|  | 0 | 2-3 | 0.004 | (0.01, 0.12) |
|  | 0 | ＞3 | 0.001 | (0.03, 0.15) |
| Trunk region | 0 | 1-2 | 0.032 | (0.003, 0.11) |
| Gynoid region | 0 | 0-1 | 0.042 | (0.001, 0.09) |
|  | 0 | 1-2 | ＜0.001 | (0.05, 0.14) |
|  | 0 | ＞3 | 0.001 | (0.03, 0.14) |
|  | 0-1 | 1-2 | 0.030 | (0.004, 0.09) |
| Corrected leg region | 0 | 1-2 | 0.024 | (0.003, 0.08) |
| Android region/ Gynoid region | 0 | 0-1 | 0.001 | (-0.10, 0.02) |
| Total Body Mass Distribution |  |  |  |  |
| Leg region | 0 | 0-1 | 0.004 | (-0.03, -0.003) |
|  | 0 | 1-2 | 0.002 | (-0.04, -0.01) |
|  | 0 | ＞3 | 0.001 | (-0.04, -0.01) |
| Corrected leg region | 0 | 1-2 | 0.045 | (-0.04, -0.0002) |
|  | 0 | ＞3 | 0.019 | (-0.04, -0.002) |
| Blood glucose(mmol/L) | 0 | 1-2 | 0.035 | (-0.95, -0.03) |
|  | 0 | 2-3 | 0.050 | (--2.24, -0.0004) |

0: Not received GAHT; 0-1: 0<Duration of treatment≤1 year; 1-2: 1 year<Duration of treatment≤2 years; 2-3: 2 years<Duration of treatment≤3 years; >3: Duration of treatment>3 years

**The criteria for delineating the various regions of the body**

The arm region is comprised of the arm and shoulder area formed by placing a line from the crease of the axilla and through the glenohumeral joint. The trunk region includes the neck, chest, abdominal, and pelvic areas. Its upper perimeter is the inferior edge of the chin and the lower borders intersect the middle of the femoral necks without touching the brim of the pelvis. The leg region includes all of the areas below the lines that form the lower borders of the trunk. The android region is the area between the ribs and the pelvis, and is totally enclosed by the trunk region. The upper demarcation is 20% of the distance between the iliac crest and the neck. The lower demarcation is at the top of the pelvis. The gynoid region includes the hips and upper thighs, and overlaps both the leg and trunk regions. The upper boundary is below the top of the iliac crest at a distance of 1.5 times the android height. The total height of the gynoid region is two times the height of the android region. Visceral adipose tissue mass and visceral fat volume were obtained using the “CoreScan” module of the enCORE software by subtracting the fat mass from the total adipose tissue in the android region on both sides of the abdominal cavity.
